# Molecular Structures of Quiescently Grown and Brain-Derived Polymorphic Fibrils of the Alzheimer Amyloid Aβ_9-40_ Peptide: A Comparison to Agitated Fibrils

**DOI:** 10.1371/journal.pcbi.1000693

**Published:** 2010-03-05

**Authors:** Chun Wu, Michael T. Bowers, Joan-Emma Shea

**Affiliations:** 1 Department of Chemistry and Biochemistry, University of California, Santa Barbara, Santa Barbara, California, United States of America; 2 Department of Physics, University of California, Santa Barbara, Santa Barbara, California, United States of America; National Cancer Institute, United States of America and Tel Aviv University, Israel

## Abstract

The presence of amyloid deposits consisting primarily of Amyloid-β (Aβ) fibril in the brain is a hallmark of Alzheimer's disease (AD). The morphologies of these fibrils are exquisitely sensitive to environmental conditions. Using molecular dynamics simulations combined with data from previously published solid-state NMR experiments, we propose the first atomically detailed structures of two asymmetric polymorphs of the Aβ_9-40_ peptide fibril. The first corresponds to synthetic fibrils grown under quiescent conditions and the second to fibrils derived from AD patients' brain-extracts. Our core structure in both fibril structures consists of a layered structure in which three cross-β subunits are arranged in six tightly stacked β-sheet layers with an antiparallel hydrophobic-hydrophobic and an antiparallel polar-polar interface. The synthetic and brain-derived structures differ primarily in the side-chain orientation of one β-strand. The presence of a large and continually exposed hydrophobic surface (buried in the symmetric agitated Aβ fibrils) may account for the higher toxicity of the asymmetric fibrils. Our model explains the effects of external perturbations on the fibril lateral architecture as well as the fibrillogenesis inhibiting action of amphiphilic molecules.

## Author Summary

Amyloid diseases are characterized by the presence of amyloid fibrils on organs and tissue in the body. Alzheimer's disease, Parkinson's diseases and Type II Diabetes are all examples of amyloid diseases. Determining the structure of amyloid fibrils is critical for understanding the mechanism of fibril formation as well as for the design of inhibitor molecules that can prevent aggregation. In the case of the Alzheimer Amyloid-β (Aβ) peptide, the structure of fibrils grown under conditions of mechanical agitation has been elucidated from a combination of simulation and experiments. However, the structures of the asymmetric quiescent Aβ fibrils (grown under conditions akin to physiological conditions) and of Alzheimer's brain–derived fibrils are not known. In this paper, we propose the first atomically detailed structures of these two fibrils, using molecular dynamics simulations combined with data from previously published experiments. In additions, we suggest a unifying lateral growth mechanism that explains the increased toxicity of quiescent Aβ fibrils, the effects of external perturbations on fibril lateral architecture and the inhibition mechanism of the small molecule inhibitors on fibril formation.

## Introduction

A number of human diseases known as amyloidoses [1],[2] are associated with the presence of amyloid plaques in organs and tissues. The main constituents of these plaques are fibrillar aggregates arising from the pathological self-assembly of normally soluble proteins. The etiology of amyloidoses is poorly understood, and the causative agents in cellular toxicity have been associated with soluble oligomers [3]–[6] as small as dimers[6], protofibrils [7]–[10] and mature fibrils[11]. The fibrillar products of aggregation (these include protofibrils as well as mature fibrils) share common structural features: they are enriched in β-sheet structure and possess a common cross-β sheet motif, in which the β-strands lay perpendicular to the main axis of the fibril [12]–[16]. In most cases, the atomic structure of the fibrils is not known, although recent computational and solid-state NMR studies have begun to provide detailed models of amyloid fibrils. [11], [17]–[26]

Perhaps the most clinically relevant amyloidosis is Alzheimer's disease (AD), the leading cause of late-life dementia. The protein implicated in AD is the 40–42 amino-acid long amyloid-β (Aβ) peptide, derived from proteolytic cleavage of the transmembrane amyloid precursor protein.[27]–[29] Experimental studies have shown that the morphology of Aβ fibrils is exquisitely sensitive to environmental conditions. Gentle mechanic shaking [11], small chemical modifications (e.g the oxidation of Met 35/M35ox[19]) or ligand binding (e.g small peptidic [30] or non-peptidic inhibitors[31]) can affect the interactions (salt bridges, hydrophobic side-chain packing etc.) between the cross-β subunits (protofilaments) constituting the fibril. This can lead to large scale changes in fibril morphology, and even to altered toxicity[11]. For instance, at pH 7.4 and 24°C, and under conditions of gentle mechanic sonication, Aβ_40_ peptides are seen to form amyloid fibrils (“agitated fibrils”) that predominantly contain 2 cross-β subunits with untwisted, “striated ribbon” morphologies. [32] Based on a combination of data from solid state NMR and scanning transmission electron microscopy (STEM), Tycko and co-workers showed that the agitated amyloid fibrils are 2-fold-symmetric (i.e have 2 equivalent cross-β-subunits). In sharp contrast, under the same solution conditions, but in the absence of sonication, the resulting “quiescently” grown Aβ_40_ fibrils predominantly contain 3 cross-β subunits with a “twisted pair” morphology. [33],[34] These quiescent fibrils appear to be more toxic than the agitated fibrils, based on studies on rat embryonic hippocampal neurons.[11] Even more striking is the fact that a slight alteration in the quiescent growth conditions leads to a different symmetry for the fibril: in one case, the 3 cross-β subunits are arranged in an asymmetric manner (2 equivalent cross-β-subunits and one 1 nonequivalent cross-β-subunit) [11], and in the other, in a symmetric manner (3 equivalent cross-β-subunits). [18] Recently, Tycko and co-workers [35] have performed solid state NMR and mass-per-length (MPL) studies on fibrils obtained from AD patients' brain extracts. These brain-seeded fibrils, which presumably reflect the relevant fibrils structures found in diseased brains, show yet another morphology, albeit one bearing strong similarities to an asymmetric quiescently grown synthetic Aβ fibril. Both predominantly contain 3 cross-β subunits that show two sets of chemical shifts for many 13C-labeled sites, and the primary difference between the synthetic quiescent and brain-derived fibrils appears to lie in the orientation of the side chains in the C-terminal β-strand of the fibril.

**Figure 1:**
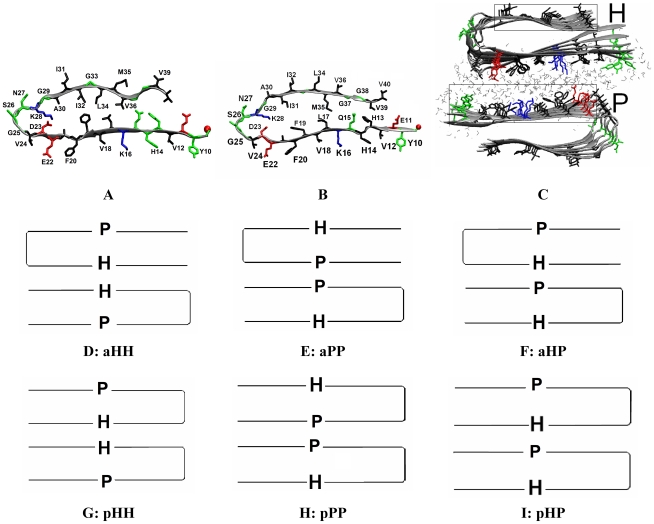
Initial structures of an Aβ_9-40_ peptide in fibrils and two cross-β subunits of synthetic fibrils. A–B: a single Aβ_9-40_ peptide in purely synthetic fibrils (A) and brain-seeded fibrils (B) consists of a hydrophobic β-strand (upper, residues 30–40), a polar β-strand (lower, residues 10–22) and a loop. Residues 1–8 are disordered and thus omitted. **C:** Starting configuration for two cross-β subunits from construct aPP (see Text S1). A cross-β subunit, containing of 6 peptides, has a hydrophobic (H) and a polar (P) surface. Only water molecules between the two interfacing surfaces (in gray triangles) are shown. Negatively charged, positively charged, polar and hydrophobic residues are colored in red, blue, green and black, respectively. N-terminal is shown in red VDW ball. D–F: Cartoons of 3 antiparallel constructs with 3 types of interfaces. G–I: Cartoons of 3 parallel constructs with 3 types of interfaces.

While experimental and computational models of the agitated fibril with 2-fold symmetry [17],[20],[22],[32] and of the quiescent fibril with 3-fold symmetry [18] have been proposed based on experimental and computational studies, there is presently no atomically detailed model of the asymmetric quiescent synthetic fibril or of the brained-seeded fibril. Based on the structure of the solved fibrils of Aβ and analysis of the experimental data for the unsolved fibrils, it emerges that all Aβ fibrils (agitated or quiescent) studied by Tycko and co-workers share the same fundamental building block: a common cross-β subunit. This subunit (shown in Fig. 1 A–B) consists of stacked β-sheets formed from the parallel in-registry assembly of a U-shaped β-strand-loop-β-strand motif. In this cross-β subunit, the β-strands are oriented perpendicular to main chain hydrogen bonding direction, with the hydrogen bonding direction laying parallel to the fibril axis. Two such cross-β subunits stack laterally (the normal direction to the β-sheet surface) to form the 2-fold symmetric agitated fibril[32], while three such units arrange in a triangle to form the 3-fold symmetric quiescent structure.[18] The atomic details of the cross-β subunits differ slightly in the agitated and quiescent models. In the quiescent asymmetric case, a slight conformational difference has been reported in the side-chains of the solvent exposed loop region (residues 23–29), but the β-sheet-to-β-sheet stacking that determines the overall morphology of the fibril is the same. In the case of the brain-derived subunits, the side-chain orientations of some of the residues are inverted with respect to those in the agitated subunit. In this study, we use the cross-β subunit of reference [32] (the structure based on the most recent refinement work by Tycko and co-workers, and one that is consistent with the original predictions of Nussinov and coworkers, ref [36]) as a starting point for our simulations. Using stacking simulations between cross-β subunits, we propose a structural model for the asymmetric quiescent Aβ fibrils and for the brain-seeded fibrils. In the case of the brain-seeded fibril, we introduce appropriate modifications (as detailed in the methods section) to capture the correct orientation of the side chains. Our simulations are akin to quiescent assembly conditions as we are not including the effects of mechanical agitation in our stacking simulations. We validate our resulting models using the experimental data provided in the work of Tycko and co-workers. [11],[35],[37],[38] We also propose a unifying lateral stacking mechanism that explains the variations in fibril's lateral architecture and toxicity under different external perturbations (mechanical shaking, M35 oxidation, and ligand binding).

## Results

### Stability of the single cross-β subunit

A starting cross-β subunit is extracted from the 2-fold symmetric model of the agitated Aβ_9-40_ fibrils. This structure corresponds to the most recent refined structure obtained by Tycko and workers [17],[32] It consists of two β-sheet layers, with each layer containing 6 Aβ_9-40_ peptides, in which each Aβ_9-40_ peptide (Fig. 1A) is arranged in a β-strand-loop-β-strand/U fold: a N-terminal β-strand (residues 10–22), a loop (residues 23–29) and a C-terminal β-strand (residues 30–40). We use the following nomenclature: since the exposed side of the C-terminal β-strand contains only hydrophobic residues (G29_I31_G33_M35_G37_V39), we refer to it as the hydrophobic “H” β-strand. In contrast, since the exposed side of the N-terminal β-strand contains hydrophobic residues separated by charged or polar residues (**Y10**_V12_H14_**K16**_V18_F20_**E22**_V24), we refer to it as the polar “P” β-strand. This nomenclature enables us to distinguish the N- and C-terminal β-strand. The implication of a uniform hydrophobic (H) surface as opposed to one interdispersed with polar residues (P) will be discussed later in the text.

We used the same cross-β subunit in modeling fibrils containing multiple cross-β subunits. It should be noted that other researchers have reported differences in the exact position of the residues in the β-sheets with the length of the β-strands sometimes changing [39]. We also note that we have treated residues 1–8 of the Aβ peptide in the fibril as disordered, based on experimental data from the studies of Tycko and coworkers. For this reason, we are only modeling residues 9–40 of Aβ in our stacking simulations. It is entirely possible that in some polymorphs these residues become structured.

Throughout this paper, we denote the cross-β subunit (Fig. 1B) as _H_U_P_ where U represents the parallel in-registry assembly of a β-strand-loop-β-strand motif, H the hydrophobic (residues 29–39) and P the polar (residues 10–22) β-sheet surfaces (Depending on the arrangement of the cross-β-subunit as part of a larger assembly, the cross-β-subunit will appear as _H_U_P_, _P_U_H_, _H_∩_P_ or _P_∩_H_).

We first considered the stability of this cross-β-subunit via four 20.0 ns long simulations at 310K. The subunit was found to be stable, as judged from the small (less than 2 Å) root mean square deviation (RMSD) from the starting structure. The fact that this subunit is stable is consistent with recent mass-per-length (MPL) data from the Tycko group in which a peak (∼9 kD/nm) corresponding to a single layer of Aβ_1-40_ is seen for the agitated and the symmetric quiescent fibril of Aβ[38] (1 subunit is ∼9 kD/nm, hence the number of subunit is equal to MPL/9). Similar studies using the new apparatus reported in reference 35 have not yet been performed on the asymmetric Aβ fibrils. The core of the 6-member cross-β-subunit (consisting of the 4 inner peptides) was very stable, while the 2 outer peptides showed more fluctuations. This is to be expected as the outer peptides have only one neighboring peptide that can provide stabilizing interactions. Since the aim of this study is to investigate lateral assembly and not on β-sheet extension of the cross-β-subunit[40], we only consider the outer peptides in the energetic, but not the structural analysis.

### Formation of a fibril containing two cross-β-subunits

Having established that the cross-β-subunit is a stable entity, we used it as a building block to construct a profibril containing two such cross-β-subunits. Several possible arrangements are possible, and we considered all 6 possibilities based on a combination of 3 interfaces and 2 orientations between two cross-β-subunits (_P_U_H_ and _H_∩_P_), as listed in Text S1. The 3 possible interfaces are HH (hydrophobic-hydrophobic), PP (polar-polar) and mixed PH (hydrophobic-polar) and the 2 possible stacking orientations are parallel (p) and antiparallel (a). Rather than starting with a pre-assembled fibril and testing its stability [21]–[24], we initiated our simulations with two separated cross-β-subunits and monitored their assembly (e.g. Fig. 1B). This enables us to study both assembly and stability.

**Figure 2:**
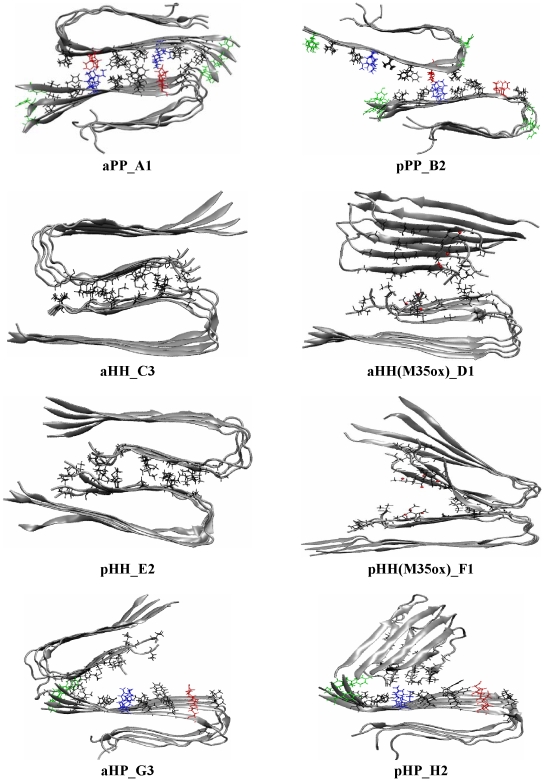
Stacking between two cross-β subunits with different interfaces at 310 K of purely synthetic fibrils. The last snapshot of a representative trajectory (out of 4 20-ns-trajectories) for each construct (see Text S1) is presented here (see Text S1 for all 4 trajectories of each construct). A1: Salt-bridge formation (E22-K16 in red and blue) between the polar surfaces of construct aPP, with the opposite charges at the interfaces. B2: Repulsion and shift between the two polar surfaces of construct pPP. C3: Ordered association of the two hydrophobic surfaces for construct aHH. D1: Partial association of the hydrophobic interface of construct aHH(M35ox) upon single oxidation of M35. E2: Ordered association of the two hydrophobic surfaces of construct pHH. F1: Partial association of the hydrophobic surfaces of construct pHH(M35ox) with single oxidation of M35. G3-H2: Disordered association between polar and hydrophobic surface of constructs aHP and pHP, respectively. Only the 4 inner strands of a cross-β subunit are shown. Negatively charged, positively charged, polar and hydrophobic residues are colored red, blue, green and black, respectively. The oxygen of the oxidized Met 35 is shown in red VDW ball.

The number of side-chain atom contacts and 6 additional structural order parameters were used to characterize the β-sheet-to-β-sheet stacking process (see Text S1). Of the 6 possible constructs, an ordered and stable fibril interface was observed only in constructs aPP, aHH, and pHH. Snapshots of the final structure from a representative trajectory for each of the 6 constructs studied shown in Figure 2. We summarize the structural features of the three ordered interfaces below, with the other three disordered interfaces described in the Supplemental Material. For **aPP** (Fig. 2-A1), the interface is stabilized by two hydrophobic pairs (F20-V18 and V18-F20, viewed from left to right), and two salt bridges (E22-K16 and K16-E22, viewed from left to right) in the cross section (along the β-sheet stacking direction) of the ordered four β-sheet layers. The side-chains at the sheet-to-sheet interface are packed head-to-head without interdigitating (“zipping”) leading to a large layer-to-layer distance (13.6±0.4 Å). In the case of **aHH** (Fig. 2-C3), a tight hydrophobic interface is formed by five hydrophobic pairs (G29-V39, I31-G37, G33-M35, M35-G32 and G37-I31, viewed from left to right) between two N-terminal β-strands (i.e. G29_I31_G33_M35_G37_V39). The lack of side-chains of the glycine residues provides a groove on one face into which the large hydrophobic side chains of the opposite cross-β unit can fit. As a result of the insertion of the large hydrophobic side chains (V39, M35 and I31) into the grooves formed by G29 and G33 on the opposite face, the resulting layer-to-layer distance (7.1±0.8 Å) in aHH is shorter than seen in aPP (13.6±0.4 Å).” In additions, the β-sheets at the interface of system aHH are slightly less twisted than those of system aPP (twist angle of ∼3° in aHH versus aPP ∼6° for aPP). For **pHH**, a tight hydrophobic interface is formed by four hydrophobic pairs (G29-I31, I31-G33, G33-M35 and M35-G37, viewed from left to right) as a result of a one-residue shift of the β-strand along the β-strand direction.

**Figure 3:**
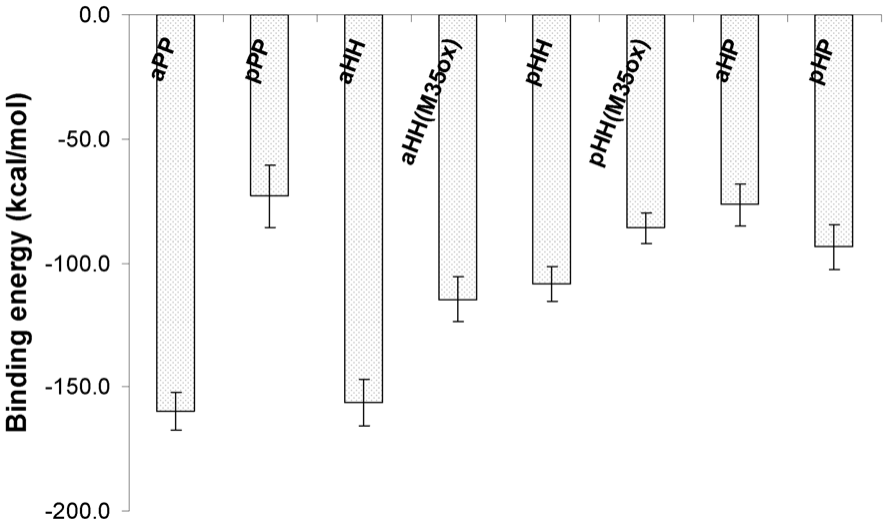
Binding energies between the two cross-β subunits with different interfaces. The binding energy was calculated over the last 5 ns simulations using the MM-GBSA implicit solvation model in the AMBER program.

In order to gain further insight into the relative stability of the fibrils with different interfaces, we calculated the binding energy between two cross-β subunits over time for each system using the MM-GBSA module in AMBER. The convergence was observed in the last 5 ns (see aHH system as an example in Text S1). The results over the last 5 ns are shown in Figure 3. A clear relative trend emerges: the ordered complexes aPP and aHH have the lowest binding energies (−159.9±7.4 and −156.2±9.5 kcal/mol respectively). The pHH construct has a less favorable binding energy (−108.4±6.8 kcal/mol) than aHH. The significant difference (∼48 kcal/mol or ∼4 kcal/mol per peptide) in binding energies between aHH and pHH illustrates that stability is not only determined by the hydrophobicity of the interface alone; the interdigitation of the side-chains at the interface also plays a key role[41]. It is interesting to note that 2D ^13^C-^13^C NMR experiments [32] have identified the presence of contact pairs I31-G37 and M35-G33 in Aβ_40_ fibrils, which further support a construct with an aHH interface over a pHH one [21],[32] in 2-cross-β-subunit fibrils. Indeed, these contact pairs are among the contact pairs (G29-V39, I31-G37, G33-M35, M35-G32 and G37-I31) present in our aHH model fibril, but not in the pHH fibril.

From an energetic perspective, aPP and aHH are the most favorable interfaces (indistinguishable within error from each other based on our binding energy calculations). However, from an entropic perspective, one could argue that the aHH interface might be slightly more favorable than the aPP interface (larger ΔS). The energetic basin associated with hydrophobic interactions (ie, the HH interface) is much broader than the narrow basin associated with distance dependent electrostatic interactions (the salt bridges at the PP interface). As a result, the HH interface can accommodate much more structural fluctuations and disorder than the PP interface. Fluctuations leading to a shifting of the two cross-β-subunits along the β-strand direction or disorder related to mis-registry can be tolerated at the HH interface, but not at the PP interface where such effects would lead to breaking of the salt-bridges and hence an overall destabilization of the fibril. As a result, the aHH interface would be the most favorable in terms of free energy, with aPP the close second. Our results are in a qualitative agreement with a recent stability studies [22]–[24] of pre-constructed 2-cross-β-subunit species of Aβ40 modeled by another popular CHARMM force field[42].

### Assembly of an asymmetric quiescent fibril containing 3-4 cross-β-subunits from 1-cross-β-subunit and 2-cross-β-subunit species

Having established that the 1-cross-β-subunit and the 2-cross-β-subunit constructs with aHH and aPP interfaces are stable, we now turn to the assembly of a larger profibril based on the 1- and 2- cross-β-subunits. In particular, we wish to construct a model for the asymmetric 3-subunit quiescent fibril seen in the experiments of Tycko and co-workers[11]. The asymmetry is suggested by the fact that two sets of chemical shifts were observed in experiment for several ^13^C-labeled sites, indicating that the sidechains of these residues are in different environments, In order to satisfy this asymmetry, two different types of interfaces between three cross-β-subunits are required. Based on our previous calculations, we expect one of the interfaces to be aHH (the most stable interface), and the other one to be aPP (the second most stable interface). The experiments of Tycko also indicate the presence of a smaller amount of a 4 cross-β-subunit fibril. Similarly to the 3 cross-β-subunit fibril, this structure will also involve the two types of interfaces.

**Figure 4:**
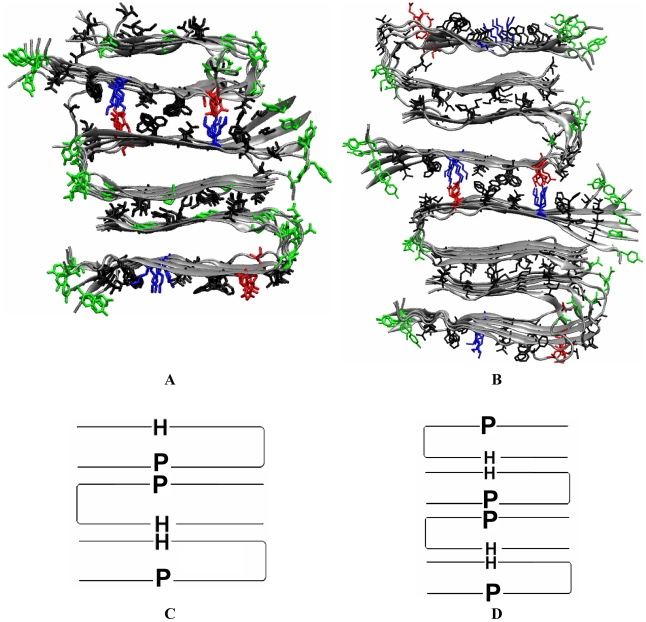
Quiescent fibril structure models of purely synthetic fibrils obtained from our stacking simulations. A: our proposed structural model of the 3-fold asymmetric quiescent Aβ40 fibrils with 3 cross-β subunits, obtained from the “2+1” stacking simulations (see all trajectories in Text S1). B: Our proposed structural model of quiescent Aβ40 fibrils with 4 cross-β subunits, obtained from the “2+2” stacking simulations (see all trajectories in Text S1). C–D: Cartoons of A and B.

A 3-cross-β-subunit protofibril can arise either from a 3 body assembly (1+1+1), or from a 2 body assembly (2+1). Here we only model the 2+1 assembly pathway, as a two-body assembly is more probable than a three-body assembly for entropic reasons. The (2+1) stacking would involve in a first step the formation of a 2-subunit fibril (_P_U_HH_∩_P_) with an aHH interface (such as the model proposed for the agitated 2-cross-β-subunit fibril). It would be followed by the lateral stacking of another cross-β-subunit such that the final fibril has 3 stacked cross-β-subunits (_P_U_HH_∩_PP_U_H_) with two interfaces aHH and aPP (Fig. 4 left). Similarly, to obtain a 4-subunit profibril, “1+1+1+1”, “2+1+1”, “2+2” (_P_U_HH_∩_P_ + _P_U_HH_∩_P_) and “3+1” (_P_U_HH_∩_PP_U_H_ + _H_∩_P_) stackings are possible. We focus our study on the aPP interface formation in the (2+2) pathway. The resulting fibril would be arranged as _P_U_HH_∩_PP_U_HH_∩_P_ with three interfaces aHH, aPP and aHH (Fig. 4 right).

We investigated the assembly of the 2+1 (_P_U_HH_∩_P_ + _P_U_H_) construct for the 3-subunit profibril and the 2+2 (_P_U_HH_∩_P_ + _P_U_HH_∩_P_) construct for the 4-subunit profibril. The simulations were initiated with the components (_P_U_HH_∩_P_ and _P_U_H_ for the 3-subunit fibril and _P_U_HH_∩_P_ + _P_U_HH_∩_P_ for the 4-subunit fibril) separated by 10 Å (∼3 water layers) along the β-sheet stacking direction. Four 20 ns simulations were performed for the 2+1 and 2+2 systems at 310 K and the formation of the aPP interface was monitored. An ordered and stable aPP interface was formed in all eight simulations (Text S1). A representative structure of the resulting 3 and 4 cross-β-subunit quiescent fibrils is shown in Figure 4. The binding energy for forming the aPP interface in the “2+1” or the “2+2” constructs was −163.4±9.9 kcal/mol, comparable to the number (−159.9±7.4 kcal/mol) seen for forming the in “1+1” aPP interface. The structural parameters are also comparable (data not shown).

Our proposed 3-cross-β-subunit asymmetric fibril (_P_U_HH_∩_PP_U_H_) structure has the following features: 1) the surface side chains of each of the 3 cross-β-subunits are not structural equivalent due to different environment (e.g either exposed to solvent or buried at the aHH or aPP interface); 2) the two exposed β-sheet surfaces of the fibril differ in hydrophobicity: one is polar/charged (exposed residues H14, K16 and E22); the other is quite hydrophobic (exposed residues I31, M35 and V39); 3) whereas the K16 and E22 residues at the aPP interface forms salt-bridges, the K16 and E22 residues at the surface are exposed to solvent and do not form salt bridges. 4) the 3 cross-β-subunits are tightly stacked and the thickness of fibril is ∼60 Å. Our proposed 4-cross-β-subunit fibril (_P_U_HH_∩_PP_U_HH_∩_P_) has two-fold symmetry and the two exposed surfaces are polar/charged. In addition, only half of the K16 and E22 residues from all 4 cross-β-subunits formed salt-bridges (those at the aPP interface). We note that Tycko and co-workers report a slight conformation difference in the side-chains of the loop region (residues 23–29) in the core cross-β-subunit between the agitated and quiescent structures. The use of the agitated cross-β-subunit as our initial building block should not affect our resulting structural model. Indeed, the loop is exposed to the solvent and plays little role in the β-sheet-to-β-sheet stacking that determines the overall morphology of the fibril. It is important to note that the loop region is highly flexible (dynamic) compared to the β-sheet regions. It is quite possible that if we ran the simulation longer, we would see some changes in the loop structure of the non-equivalent cross-β-subunit that experiences a different environment from the one seen in the symmetric agitated fibril.

### Stability of brain-seeded fibril containing 3 cross-β-subunits

**Figure 5:**
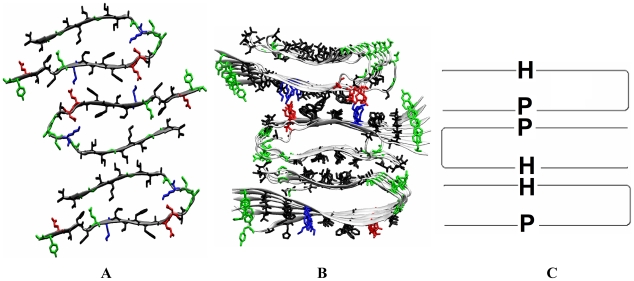
Quiescent fibril structure model of brain-seeded fibrils. A: An initial structure B–C: a representative last snapshot in ribbon and in cartoon. The fibrils contain three cross-β subunits from four 20-ns stability simulations (see all trajectories in Text S1).

Recent experiments by Tycko and co-workers [35]on brain-seeded Aβ fibrils indicate that these fibrils bear strong morphological resemblance to the quiescently grown asymmetric synthetic fibrils. Both chemical shifts and dipole-dipole couplings [35]show the peptide in brain-seeded fibrils adopts the same β-strand-loop-β-strand conformation as in the asymmetric quiescent fibrils (e.g. F19, A30, I31, L34 and M35 in β-strands; D23, V24 and G25 in non-β-strand conformation; presence of a D23-K28 salt bridge). MPL data indicate that the brain-seeded structures (again like the quiescent structures) consist primarily of fibrils with 3 cross-β subunits and NMR experiments show two sets of chemical shifts for many 13C-labeled sites. The primary difference between the brain-seeded and asymmetric quiescent fibrils lies in the orientation of the side-chains. 2D radiofrequency-assisted diffusion (RAD) spectra[35] indicate an additional F19-I31 side chain – side chain contact, suggesting the side chains in the C-terminal β-strand are “up-down” flipped as compared with the asymmetric quiescent fibrils. This flipping could be enabled by the flexible backbone of G29 residue, which could accommodate either orientation of side-chains in the C-terminal β-strand. Using the 3-fold asymmetric quiescent fibril model as a template, we construct a model for the brain-seeded fibril by flipping the side chains at the C-terminal β-strand (Fig. 5). The 3 cross-β-subunit model has both aHH and aPP interfaces. While the interactions at the aPP interface are the same as in the asymmetric quiescent fibrils, the detailed interactions at the aHH interface is changed as the sidechains are flipped (i.e the side chains of I32, I34 and V36 now interdigitate). The stability of our brain-seeded fibril model was confirmed by four 20.0 ns MD simulations at 310K in which the brain-seeded fibril was found to be stable, as judged from the small (less than 2 Å) root mean square distance (RMSD) from the starting structure. The binding energies for forming the aHH and aPP interfaces are respectively −155.4±5.9 and −160.4±7.9 kcal/mol, which are comparable to those in the synthetic fibrils with 3-cross-β-subunits.

### Destabilizing effect of M35ox on the hydrophobic interface (HH) of synthetic fibrils

Much like mechanical agitation, the chemical oxidation of M35 can dramatically alter fibril lateral formation. In the case of the Aβ42 peptide, the β-sheet-to-β-sheet stacking process is completely blocked such that the resulting Aβ42(M35ox) fibril contains only a single cross-β-subunit [19]. Since both the Aβ40 and the Aβ42 cross-β-subunits contain similar β-strand-loop-β-strand motifs (they differ in the precise location of the loop), one would expect the M35 oxidation to affect Aβ40 fibrils in a similar manner as Aβ42. The structure of the M35ox variant of Aβ_9-40_ has not been solved experimentally. Here, we consider a M35ox variant of Aβ_9-40_ and investigate the effects of the oxidation, first on the single cross-β-subunit, then on the assembly (monitored by stacking simulations) of the aHH and pHH constructs.

We find that the stability of the 1-cross-β-subunit in our simulations is not affected by the single oxidation of M35, likely a result of the fact that the side chains of the Met residues are exposed to the solvent and hence do not contribute to the stability of the cross-β-subunit. In contrast, the “1+1” assembly simulations (with the M35 oxidation) show a reduction in the number of trajectories that lead to an ordered assembled complex (from 4 to 2 for aHH and from 3 to 1 for pHH out of a total four trajectories for each construct) (see Text S1). This confirms that hydrophobic interactions play an important role in stabilizing the pHH and aHH interfaces. Introduction of a polar side-chain at the interface level (here via single oxidation of the hydrophobic M35 residue) significantly affects the formation of the hydrophobic interfaces. The stronger hydration tendency of the M35ox residues in the aHH(M35ox) and pHH(M35ox) constructs in comparison to the M35 residues in constucts aHH and pHH is directly supported by the presence of more water molecules in the first solvation shell (<2.8 Å) of these side chains, averaged over the last ns of the simulations (Table 2). ∼22 and ∼28 waters are present in systems aHH(M35ox) and pHH(M35ox), respectively, while only ∼10 and ∼12 waters are present for systems aHH and pHH, respectively. Binding energy calculations also reveal a weaker binding energy (less favorable binding) between the two cross-β-subunits in constructs aHH(M35ox) and pHH(M35ox) than that in constructs aHH and pHH by ∼41.4 and ∼22.6 kcal/mol, respectively (See Fig. 3). Again, our finding is in a qualitative agreement with a recent stability study [22] of pre-constructed 2-cross-β-subunit species of Aβ40 M35ox mutants modeled using the CHARMM force field[42]. We predict that Aβ40 M35ox mutants would predominantly exist in a single layer structure. It would be interested to see MPL data on this system to confirm this prediction.

## Discussion

Amyloid fibrils are often generated via mechanical agitation in the laboratory, as this process speeds up fibril formation. Fibril formation in the brain, however, more likely resembles quiescent conditions. Indeed, MPL measurements performed by Tycko and co-workers [35] have recently shown that fibrils seeded from Alzheimer's brain-derived fibrils (likely reflecting the relevant structures present in AD brains) adopt a structure that has higher similarity to quiescent synthetic fibril structures[11] (a 3 cross-β subunit structure) than to agitated fibrils (a 2 cross-β subunit structure). Furthermore, the brain-seeded fibrils show much greater morphological similarities to the asymmetric quiescent fibril structure than to the symmetric quiescent polymorph, presumably because more perturbations were involved in the seeding and growth procedure that generated the fibrils with symmetric structure. [18] Structures have been proposed for both the 2-fold agitated fibrils [20],[23],[32] and for the 3-fold symmetric, quiescently grown fibrils [18]. In both cases, the fundamental building block is the same cross-β subunit consisting of stacked β-strand-loop-β-strand motifs (see Figure 1). In the agitated fibril, two such cross-β-subunits are stacked laterally. In the symmetric quiescent structure, 3 cross-β-subunits are arranged in a triangular configuration. The atomistic structure of the asymmetric 3-unit quiescent fibril and the brain-seeded fibril, on the other hand are not known.

In the present work, we propose the first atomistic structure for the asymmetric 3-subunit quiescent synthetic fibril using molecular dynamics that probe the assembly of the core cross-β subunits. This structure is then used as a template for a brain-seeded model that differs primarily from the synthetic quiescently grown fibrils in the orientation of the side chains at the C-terminal β-strand. Our simulations suggest that the asymmetric quiescent fibrils contain 3 cross-β subunits arranged in 6 tightly stacked β-sheet layers (_P_U_HH_∩_PP_U_H_) with two interfaces aHH and aPP (Fig. 4 left).

Our proposed structural model is consistent with the known constraints experimentally identified by Tycko and coworkers [11],[32],[43]. The experimental observations are the following: (A) the quiescent fibrils share similar cross-β subunit with the agitated fibril; (B) the quiescent fibrils predominantly contains 3 cross-β-subunits rather than the 2 cross-β-subunits seen in the agitated fibrils (this information is obtained from analysis of the mass per length (MPL) values from STEM experiments); (C) the quiescent fibril contains two structurally equivalent and one structurally non-equivalent parts. This conclusion is drawn from the fact that many residues exhibit two sets of ^13^C chemical shifts, with an approximate 2∶1 ratio of NMR signal intensities. In particular, splitting of I31 was observed even after three generations of the quiescent fibrils (Fig. 2 of Ref. 10). (D) Partial occupation of an intermolecular K16-E22 salt bridge. Tycko and co-workers report the presence of dipole-dipole couplings between side-chain C_σ_ carbons of E22 residues and side-chain N_ζ_ nitrogens of K16 residues in quiescent fibrils, but not in agitated fibrils [11].

Our proposed structure clearly satisfies constraints A and B. Constraint C is satisfied as well: Our model (See Fig. 4 left) contains two structurally equivalent and one structurally non-equivalent parts (_P_U_HH_∩_PP_U_H_): 2 equivalent layers at the interfaces (aHH and aPP) and one non-equivalent outer sheet-layer exposed to solvent (P and H). Hence, the side chains on the peptide surface (Residues H14, K16, V18, F20, E22, and V24 of the polar β-strand/P and I31, M35 and V39 of the hydrophobic β-strand/H) would experience two chemical environments with a ratio of 2∶1, consistent with the experimentally observed chemical shift splitting with a ratio of 2∶1. As a specific example, we turn to residue I31 for which two sets of ^13^C chemical shifts, with an approximate 2∶1 ratio of NMR signal intensities, are observed experimentally. The implication is that this residue is found in two difference chemical environments. This is consistent with our three-layer asymmetric structure. One environment corresponds to the I31 residues being buried at the interface; the second corresponds to the I31 residues being exposed to the solvent. There are two instance where the I31 is buried, and one where it is exposed, corresponding to the experimentally observed 2∶1 splitting ratio. In terms of constraint D, our construct indeed shows partial occupancy of the K16-E22 salt bridge. K16-E22 salt bridges are formed at the aPP interface between the upper two sheet-layers (_P_U_HH_∩_PP_U_H_). The K16 and E22 salt bridges on the outer polar surface are still exposed to water, leading to a 2/3 occupancy of the K16-E22 salt bridges (See Fig. 4 left).

It is important to note that the observation of multiple sets of NMR signals for a single labeled site in the fibril (as seen in the experiments of Tycko cite) does not rule out the presence of a co-existing population of symmetric structures along with asymmetric structures. Indeed, an alternate explanation for multiple sets of NMR signals is that the sample in reality contains a mixture of fibrils (e.g different symmetric and asymmetric morphologies). However, one can argue in the case of the quiescently grown fibrils of reference[11],[37] that the presence of both a K16-E22 salt bridge coupled to the presence of a 3 cross-β unit structure sufficiently implies that even in a polymorphic sample, the asymmetric structure would be the major species. The brain-seeded fibrils have not been characterized to the extent of the quiescent fibrils and many more NMR contacts remain to be established. Further experimental data for the brain-seeded fibrils (for instance, a clear signature of a K16-E22 salt bridge at the aPP interface and further contacts at the aHH interface) are required to fully validate our brain-seeded model. At present, the experimental data does not seem consistent with a symmetric 3 cross-β unit fibril as a major species, although such polymorphs may be present in the brain. It is important to note that the final morphology of a fibril is dictated by both thermodynamic and kinetic factors. The data of the 2005 Tycko paper [11] (reporting the asymmetric structure) and 2008 paper (reporting the symmetric structure) [18] pertain to fibrils grown under different conditions. It is apparent that the symmetric “triangle” structure cannot be energetically more stable that the asymmetric 3-layer structure, given the fact that there are far fewer hydrophobic contacts between the subunits. Entropically, the formation of the symmetric structure (if one considers that it forms from pre-formed subunits, which may not be the case), would have to occur in a concerted 3-body 1+1+1 manner. An “open” 1+1 complex on its own would likely not be stable (or at least not as stable as a closed stacked form). Thermodynamically, the stacked asymmetric structure is certainly going to be favored, with the symmetric structure likely a result of kinetic trapping during the experimental procedure. It is compelling to note that the brain derived structure, one that has formed slowly in the brain, perhaps even over decades (ie that had more opportunity to find a thermodynamically stable structure), does not appear to be consistent with the triangle structure, but rather with a layered structure.

**Figure 6:**
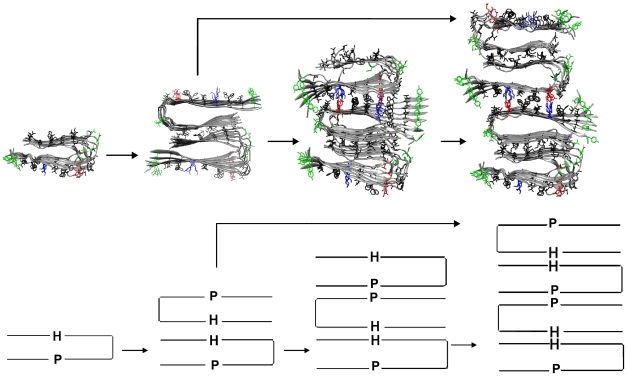
Lateral growth mechanism in the formation of a multiple layer protofibril.

Our stacking simulations enable us to propose a lateral growth mechanism for the formation of a multiple layer protofibril (<24 peptides). This protofibril acts as a seed for the growth of mature fibrils by the addition of peptides to the two edges (via the nucleation-growth mechanism[1]). This mechanism is shown in Figure 6. In the first step, a 2-cross-β-subunit protofibril is assembled from two 1-cross-β-subunit protofibrils[38] by forming an aHH interface, which is stabilized by hydrophobic and van der Waals (VDW) interactions via interdigitation of the facing side chains. In the second step, the 2-cross-β-subunit protofibril with an aHH interface further assembles with another 1-cross-β-subunit into a 3-cross-β-subunit protofibril. The new interface aPP is stabilized by salt bridges, hydrophobic and VDW interactions. Growth to a 4-cross-β-subunit protofibril is possible, following one of two 2-body assembling pathways: formation of an aPP interface between two 2-cross-β-subunit protofibril (2+2) or formation of an aHH interface by adding a cross-β-subunit on top of 3-cross-β-subunit protofibril (3+1). Further lateral growth into larger (5 or greater) cross-β-subunit complexes is likely limited by the twisting of the β-sheet-layer and other structural defects in the cross-β-subunit which prohibits subunit-to-subunit stacking. In other words, the lateral growth is limited by a faster increase of the entropic cost (i.e. fast decrease of translation, rotation and conformation entropy upon stacking) than the increase of the favorable interactions. In fact, a maximum of 4 peptide layers/cross-β-subunits in the fibril is seen experimentally, as opposed to the ∼10^3^ peptide repetition along the fibril axis for a ∼µm length fibril.

This lateral growth mechanism explains the effects of external perturbation on synthetic fibril formation. For example, mechanical shaking of the solution kinetically blocks the formation of the aPP interface (which is less stable than aHH interface) probably induced by the air water surface. It would hinder the formation of a 3 or 4 cross-β-subunit leaving the 2-cross-β-subunit protofibril (with aHH interface) for growing mature 2-cross-β-subunit fibril as the major product. This is consistent with the experimental observation that under conditions of mechanical agitation, the predominant product is a 2-cross-β-subunit fibril. Another example is a chemical perturbation via oxidation that affects the structure of the Aβ fibrils. Our simulations suggest that, much as is the case for the Aβ42(M35ox) fibrils [19], the Aβ40(M35ox) fibrils would exist predominantly in a single cross-β-subunit form. The oxidation significantly destabilizes the aHH interface (we see disordered stacking trajectories and a weaker binding energy in system aHH(M35ox) and pHH(M35ox). This prevents the formation of multiple (>2) cross-β subunit fibrils, hence leading to predominance of a 1-cross-β-subunit fibril.

It is tempting to speculate about why asymmetric quiescent fibrils are more toxic than agitated fibrils. Although the precise mechanism of toxicity of fibrils and early aggregates is still a matter of debate, it is likely that the exposure of hydrophobic side chains, normally buried in a folded protein or dispersed in an unfolded ensemble, is a key component in toxicity [1]. In the most stable 2-cross-β-subunit fibrils aHH (_P_U_HH_∩_P_) (the most likely candidate for the structure of the agitated fibril), the continuous hydrophobic surfaces are buried, with the exterior sheet-layers hydrophilic. The solvent exposed surface of the 2-subunit (_P_U_HH_∩_P_) fibril (e.g **Y10**_V12_H14_**K16**_V18_F20_**E22**_V24 **of** the N-terminal β-strand), with small hydrophobic patches interdispersed with non-polar residues, resembles the surface of a folded protein. In contrast, our proposed asymmetric quiescent Aβ_40_ fibrils with 3 cross-β-subunits (_P_U_HH_∩_PP_U_H_) has a (large) exposed continuous hydrophobic face (-U_H_) to the solvent (i.e. G29_I31_G33_M35_G37_V39 of the C-terminal β-strand). This surface may interfere with the normal function of other proteins possibly by binding to and disabling them. In the same spirit, the 1-cross-β-subunit fibril with the M35ox substitution also has a large exposed hydrophobic surface, which may also be one of the factors responsible for the higher toxicity of the Aβ_42_(M35ox) fibrils [44] over the wild type fibrils.

If indeed having a the large exposed hydrophobic surface of fibrils leads to higher toxicity, then “detergent-like” ligands may provide an effective therapeutic for amyloidoses: they could be used to cover the hydrophobic surface by binding their hydrophobic part to the hydrophobic surface, thus exposing their hydrophilic part to the solvent. The exposed hydrophilic part would help improve the solubility of the protofibrils. In addition, these ambiphilic ligands might also cap the lateral growth of protofibrils by blocking the formation of the aHH interface. This may explain the mode of action of both a novel class of peptidic inhibitors designed by Soto et al. [30] and a weaker non-peptidic inhibitor (Congo red) [45], both of which exhibit this ambiphilic feature (hydrophobic/aromatic side chains on one face; hydrophilic on the other).

## Methods

### 

#### System preparation

A neutral simulation system consists of 1–4 cross-β-subunit (6 Aβ_9-40_ peptides per cross-β-subunit), 6–24 sodium ions and ∼5,000–13,000 water molecules (see Table 1 for details). The Duan *et al* all-atom point-charge force field[46] (AMBER ff03) was chosen to represent the peptide. The parameters for single oxidized Met were derived by following the same protocol used in developing AMBER ff03. The solvent was explicitly represented by the TIP3P [47] water model. An Aβ_9-40_ peptide in the cross-β-subunit has a β-strand-loop-β-strand configuration with a “polar” β-strand spanning residues 10–22, a loop spanning residues 23–29 and a hydrophobic β-strand spanning residues 30–40 (See Fig. 1A). The initial structure of the 1-cross-β-subunit (See Fig. 1C), containing 6 peptides, is a computationally refined structure based on the experimental constraints, which is the latest structure provided by Tycko and coworkers[32],[35]. The dimensions of the 1-cross-β-subunit are 30×50×16 Å^3^ along, respectively, β-sheet extension direction (main-chain hydrogen bond direction), β-strand direction and β-sheet stacking direction/lateral direction (perpendicular to the β-sheet surface). For a 1-cross-β-subunit, both wild type methionine (M35) and its singly oxidized form (M35ox) were studied. For 2-cross-β-subunit systems, two 1-cross-β-subunits were aligned and separated by 10Å (∼3 water layers) along the β-sheet stacking direction (Fig. 1B), allowing for optimal binding while largely reducing the high computational cost for the diffusion step of the binding process. A 1-cross-β-subunit has a hydrophobic β-sheet surface (H) and a “polar” β-sheet surface (P). Based on the possible 2 relative orientations (parallel or anti-parallel) and 3 types of interfaces (PP, HH and HP; H: hydrophobic; P: polar), a total of six systems were constructed, leading to six possible β-sheet-to-β-sheet interfaces (aPP, pPP, aHH, pHH, aHP and pHP in Text S1). To examine the role of the oxidized M35 on β-sheet-to-β-sheet stacking, the singly oxidized M35 (M35ox) was considered in the two systems (aHH and pHH) in which the hydrophobic β-sheets were facing each other (Text S1). For the 3 and 4 cross-β-subunit systems, 2+1 and 2+2 systems (with the aHH interface in the 2-cross-β-subunit fibril) were simulated. 6–24 positive sodium ions (Na+) were added to neutralize the 6–24 negative charges carried by 6–24 Aβ_40_ peptides. For the brain seeded fibril system, a 3 cross-β-unit was preformed (Fig. 5 right). The solute molecules were immersed in a rectangle box of ∼5,000–13,000 water molecules with dimensions of ∼50-92×82-92×44-117 Å^3^. The periodic water box was constructed in such a way that the solute was at least ∼10 Å away from the box surface and the minimum distance between the solute and the image was ∼20 Å.

**Table 1:** Simulated systems (quiescent conditions).

System ID	Content		Lateral stacking interface		Num of sim.	Length of each (ns)
		orientation*	hydrophobicity†	separation(Å) ‡		
1-subunit	1 subunit: 6×Aβ_9-40_	-	-	-	4	10
1-subunit(M35ox)	1 subunit: 6×Aβ_9-40_(M35ox)	-	-	-	4	10
aPP	1 subunit +1 subunit	anti-parallel	_H_U_P_-_P_∩_H_	10	4	20
pPP	1 subunit +1 subunit	parallel	_H_U_P_-_P_U_H_	10	4	20
aHH	1 subunit +1 subunit	anti-parallel	_P_U_H_-_H_∩_P_	10	4	20
aHH(M35ox)	1 subunit(M35ox) +1 subunit(M35ox)	anti-parallel	_P_U_H_-_H_∩_P_	10	4	20
pHH	1 subunit +1 subunit	parallel	_P_U_H_-_H_U_P_	10	4	20
pHH(M35ox)	1 subunit(M35ox) +1 subunit(M35ox)	parallel	_P_U_H_-_H_U_P_	10	4	20
aHP	1 subunit +1 subunit	anti-parallel	_P_U_H_-_P_∩_H_	10	4	20
pHP	1 subunit +1 subunit	parallel	_P_U_H_-_P_∩_H_	10	4	20
3-subunit	2 subunit +1 subunit	anti-parallel	_P_U_HH_∩_P_-_P_U_H_	10	4	20
4-subunit	2 subunit +2 subunit	anti-parallel	_P_U_HH_∩_P-P_U_HH_∩_P_	10	4	20
brain-seeded 3-subunit	3 brain seeded subunits	-	_P_U_HH_∩_PP_U_H_	-	4	20

*a/p: the loop region of the one U-shape layer is anti-parallel/parallel to the loop region of the other subunit at the stacking interface (Fig. 2 A1 and E2).

†Polar b-sheet surface (P): 6 polar b-strands (residues 10–22); Hydrophobic b-sheet surface (H): 6 hydrophobic b-strands (residues 30–40).

‡The two interfacing surfaces are well aligned and separated by ∼3 water layers along the stacking direction.

**Table 2:** Number of water in the first solvation shell (<2.8 Å) of M35/M35ox residues on the 8 inner strands of 2-cross-β-subunit.

ID/Traj.	1	2	3	4	Average
1-subunit	11±3	11±3	11±2	11±3	22†
1-subunit(M35ox)	16±2	17±3	17±2	17±2	34†
aHH	11±3	10±2	9±2	10±2	10
aHH(M35ox)	29±4	20±2	19±3	22±3	22
pHH	20±3	10±1	15±2	4±2	12
pHH(M35ox)	30±4	36±4	26±4	22±2	28

*Averaged over the last ns.

†scaled doubly to 8 Met residues of a 2-subunit.

#### MD simulation

The AMBER 9 simulation package[48] was used in both molecular dynamics simulations and data processing. The system was subjected to periodic boundary conditions. After an initial energy minimization, a total of 52 simulations (4 runs for each of 13 systems) were performed with different initial random velocities. The initial velocities were generated according to the Maxwell-Boltzmann's distribution at physiological temperature (310 K). A short 1.0 ns molecular dynamics at 310 K in the NPT ensemble (constant pressure and temperature) was performed to adjust system size and density, and to equilibrate the solvent. The simulations were continued at 310K for 9/19 ns in the NVT ensemble (constant volume and temperature). The particle-mesh Ewald method [49] was used to treat the long-range electrostatic interactions. SHAKE [50] was applied to constrain all bonds involving in hydrogen atoms and a time step of 2.0 fs was used. Non-bonded forces were calculated using a two-stage RESPA approach [51] where the forces within a 10 Å radius were updated every step and those beyond 10 Å were updated every two steps. Temperature was controlled at 310K using the Berendsen algorithm [52] with a coupling constant of 2.0 ps. The center of mass translations and rotations were removed every 500 steps. Studies have shown this removes the “block of ice” problem. [53],[54] The trajectories were saved at 2.0 ps intervals for further analysis.

#### Binding energy calculation

The binding energy for a complex was evaluated for the snapshots in the last 5 ns of each system using the MM-GBSA (Molecular Mechanics-Generalized Born/Surface Area) module [55] in the AMBER package. In MM-GBSA, the solvation free energy is represented by a Generalized Born term (the electrostatic part of the solvation) plus a Surface Area term (the apolar part of the solvation free energy). Although the MM-GBSA calculations may overestimate the absolute binding energy as a result of missing entropic terms of solute(such as conformational entropy change of the solute upon binding and etc.), they usually provide a reasonable estimate on the relative binding energy when the entropic parts of the two systems are comparable.[55],[56]

### Six order parameters for β-sheet-to-β-sheet stacking

As the 1-cross-β-subunit is stable and rigid, we can define a local coordinate system as follows: The origin is set to the center-of-mass (COM) of the interfacing sheet-layer of the two sheet-layers for 1-cross-β-subunit; the three coordinates are along the β-sheet extension direction, β-strand direction and β-sheet stacking direction (perpendicular to the β-sheet surface). Hence six parameters (*α*, *β*, *γ*, *a*, *b* and *c*) are used to characterize the structural relationship (rotation and translation) between two interfacing β-sheet-layers of the two 1-cross-β-subunits under a rigid body assumption: *α*, *β* and *γ* are the rotation angles of the β-sheet extension, β-strand and β-sheet stacking directions, respectively and (*a*, *b* and *c*) are translation distances along the three directions, respectively. The β-strand direction is defined by the direction of the third or fourth β-strand in the interfacing β-sheet-layer of the 1-cross-β-subunit. The β-sheet direction is defined by the same residues (Cα atoms) of the second and fifth β-strands; and the β-sheet stacking direction is obtained by the cross-product of the first two directions.

## Supporting Information

Text S1 Analysis of the stacking and stability simulation data.
(2.90 MB PDF)
