# Supplementary material for: Molecular Structures of Quiescently Grown and Brain-Derived Polymorphic Fibrils of the Alzheimer Amyloid Aβ9-40 Peptide: A Comparison to Agitated Fibrils
Source: PLoS Comput Biol. 2010 Mar 5;6(3):e1000693. doi: 10.1371/journal.pcbi.1000693 (PMC2832665; doi:10.1371/journal.pcbi.1000693)
Supplement: Text S1 — Analysis of the stacking and stability simulation data. (2.90 MB PDF) [file pcbi.1000693.s001.pdf]

Supporting material for "*Molecular structures of quiescently-grown and brain-derived polymorphic fibrils of the Alzheimer Amyloid A$\beta_{9\text{-}40}$ peptide and A comparison to agitated fibrils***" by Wu et al.**

Stacking analysis summary:

The number of side-chain atom contacts between the two cross-β subunits was used to characterize the β-sheet-to-β-sheet stacking process (see supplementary material Fig. S9-S12). Constructs (aPP, aHH and pHH) equilibrated within 5.0 ns (based on monitoring of the atomic contacts), while (pPP, aPH and pPH) only started reaching a plateau around 15.0 ns, with only a partial number of the total possible number of contacts reached. The development of the 6 order parameters defined above in all 32 trajectories (4 for each system) are shown in Figures S13-S20 of the supplementary material, with their average value over the last ns tabulated in Table S1. Based on these structural parameters and the number of side-chain atom contacts, averaged over the last 1.0 ns, we classify a 2- cross-β-subunit fibril as stable and ordered if the atom contacts exceed 400, all rotation angles are less than 40º and the translation distances are less than 18 Å. With these criteria, an ordered, stable fibril was formed in the majority (3, 4 and 3 out of 4) of trajectories for the aPP, aHH and pHH constructs, respectively, whereas almost no ordered, stable fibrils were formed for the pPP, aHP and pHP constructs (i.e 0, 0 and 1 out of 4 trajectories, respectively). The stable constructs aHH and pHH were stabilized almost entirely by atom contacts between hydrophobic residues, while the atom contacts for aPP were split between hydrophobic contacts and salt bridges.

Structural features for construct pPP, aHP and pHP:

The final frame from a representative trajectory for the 3 disordered interfaces illustrates the major structural features of each final 2-subunit complex (Fig. S2, S7 and S8). For pPP, charge-charge repulsion (K16-K16 and E22-E22, viewed from left to right) leads to the relative shift between two subunits (Fig S2 B2). For the

mixed interfaces, both aHP and pHP arrangements are unstable. Only partial contacts between the two layers are formed and the side chains of the charged residues (E22 and K16) remain exposed to solvent. The mixed HP stacking is likely unstable because of the desolvation cost associated with the polar/charged sheet-layer surface.

Table S1 Side-chain atom contacts (cutoff 4Å), relative rotation (α, β, γ) in degree, translation (a, b, c) in Å between two interfacing β-sheets (See Method for the definitions) in 4 trajectories for each construct (averaged over the last 1 ns in Fig. S9-S20).

traj. \ ID		aPP	pPP	aHH	aHH(M35ox)	pHH	pHH(M35ox)	aHP	pHP
1	Atom contacts	940	***87****	536	491	***261***	***302***	***243***	***391***
	α	9	125	6	***71***	28	23	25	39
	β	11	78	6	33	98	78	13	42
	γ	4	82	7	62	97	76	26	15
	a	1.9	2.7	3.2	13.3	6.1	11.4	6.4	13
	b	9.8	22.9	17.7	11.3	24.1	19.1	9.67	3.8
	c	13.2	30.4	7.1	10.6	14.7	11.2	18.1	16.5
2	Atom contacts	986	***390***	641	533	528	***251***	***210***	***296***
	α	9	8	26	14	7	15	51	72
	β	10	23	21	14	20	81	30	75
	γ	11	20	7	10	21	80	40	25
	a	0.4	10.2	5.5	9.3	1.6	6.8	20	9.9
	b	7.1	20.2	13.3	9.2	4.1	20.1	4.5	2.6
	c	14.3	17.9	6.9	8.5	11.1	15.5	9.6	12.2
3	Atom contacts	583	***351***	606	438	410	***209***	***382***	***397***
	α	7	74	26	32	19	15	29	52
	β	***47***	35	23	28	36	57	40	38
	γ	47	72	11	22	*38*	57	39	32
	a	0.6	1.9	6.7	11.4	2.1	5.3	1.6	14.9
	b	18.1	16.5	12.5	***20.2***	10.5	15.2	7.9	14.8
	c	14.1	22.4	6.3	11.7	12.5	14.7	21	19.2
4	Atom contacts	975	***174***	584	416	471	471	***304***	442
	α	6	17	36	21	22	13	101	6
	β	8	56	*37*	19	27	22	99	24
	γ	5	57	12	20	35	22	26	25
	a	0.4	4.1	9.6	5.3	9.1	5.8	8.7	9.5
	b	8.2	31.1	13.5	10.4	6.9	9.2	13.1	10.1
	c	13.5	23.7	6.7	10.9	8.1	12.1	11.9	12.4
sum	Ordered stacking	3	0	4	2	3	1	0	1

* itatic bold: indicator of disordered stacking

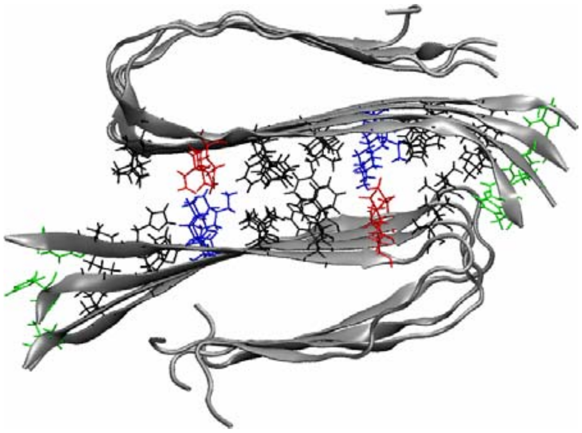

aPP_A1

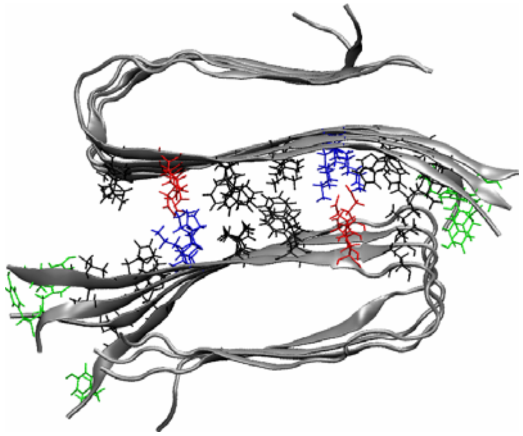

aPP_A2

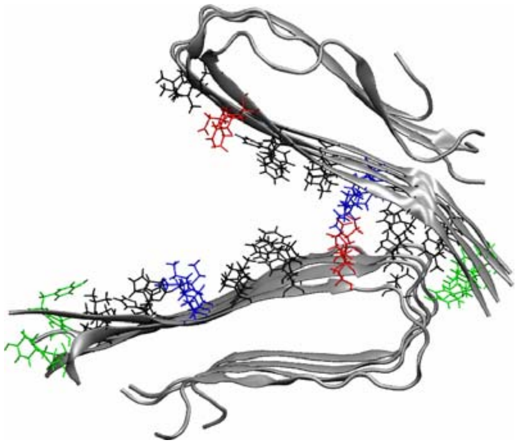

aPP_A3

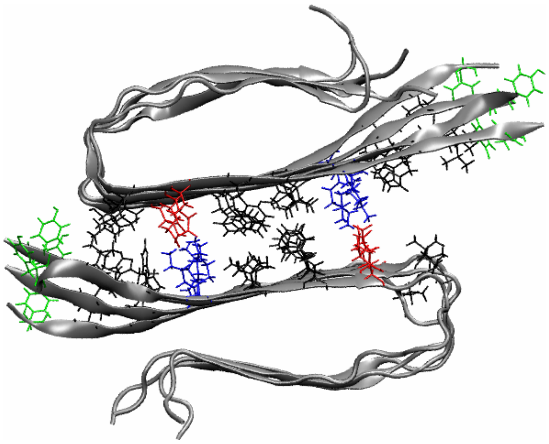

aPP_A4

Figure S1 The last snapshots from 4 trajectories of construct aPP (see Table 1). Only inner 4 strands of a subunit are shown. Negatively charged, positively charged, polar and hydrophobic residues are colored red, blue, green and black, respectively.

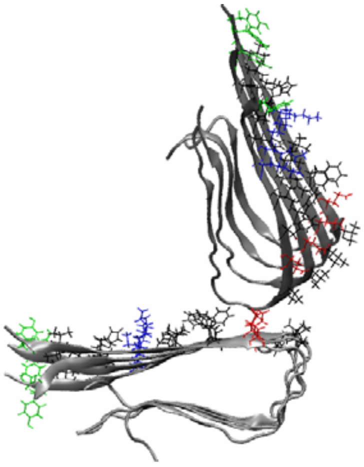

pPP_B1

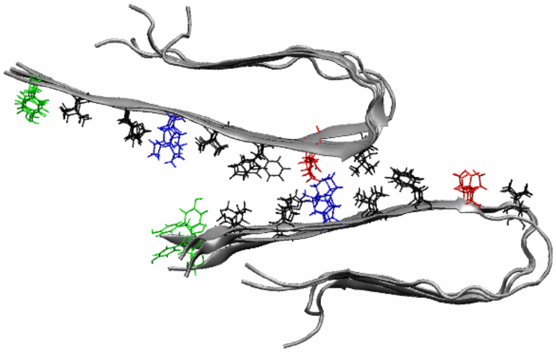

pPP_B2

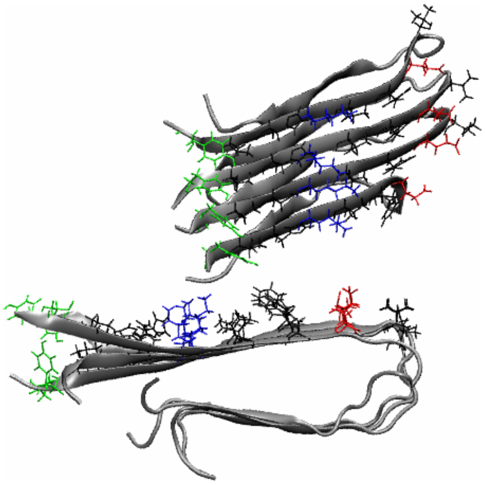

pPP_B3

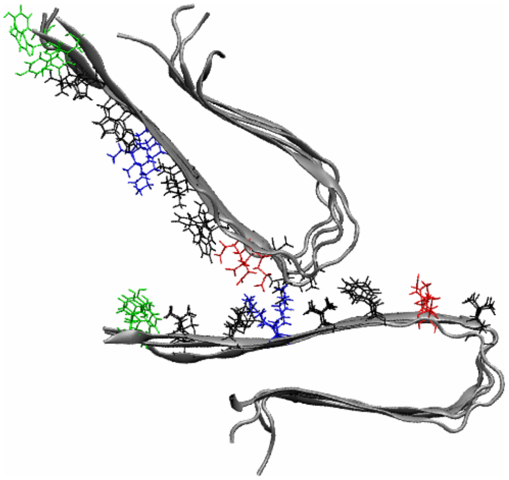

pPP_B4

Figure S2 The last snapshots from 4 trajectories of construct pPP (see Table 1). Only inner 4 strands of a subunit are shown. Negatively charged, positively charged, polar and hydrophobic residues are colored red, blue, green and black, respectively.

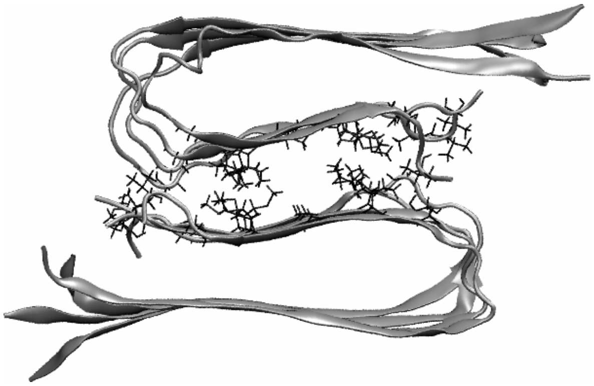

aHH_C1

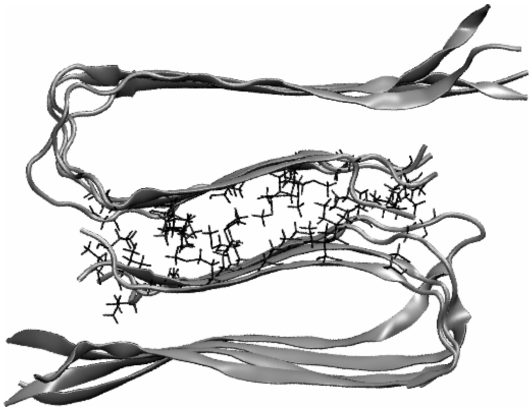

aHH_C2

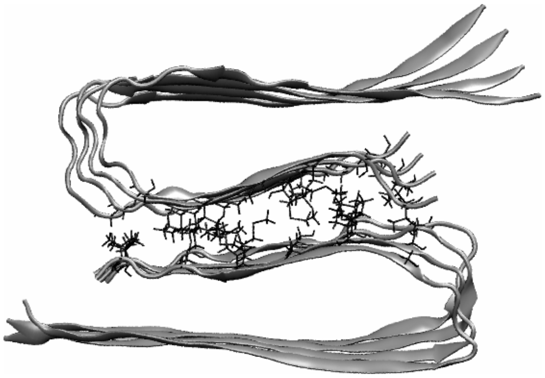

aHH_C3

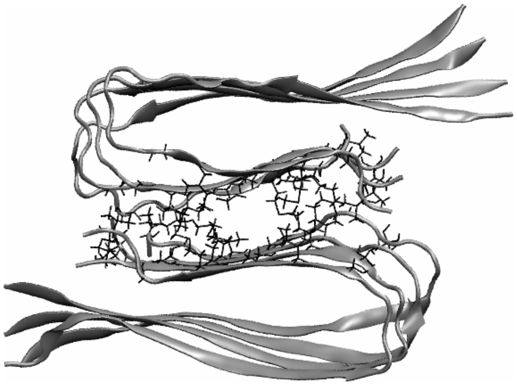

aHH_C4

Figure S3 The last snapshots from 4 trajectories of construct aHH (see Table 1). Only inner 4 strands of a subunit are shown. Negatively charged, positively charged, polar and hydrophobic residues are colored red, blue, green and black, respectively.

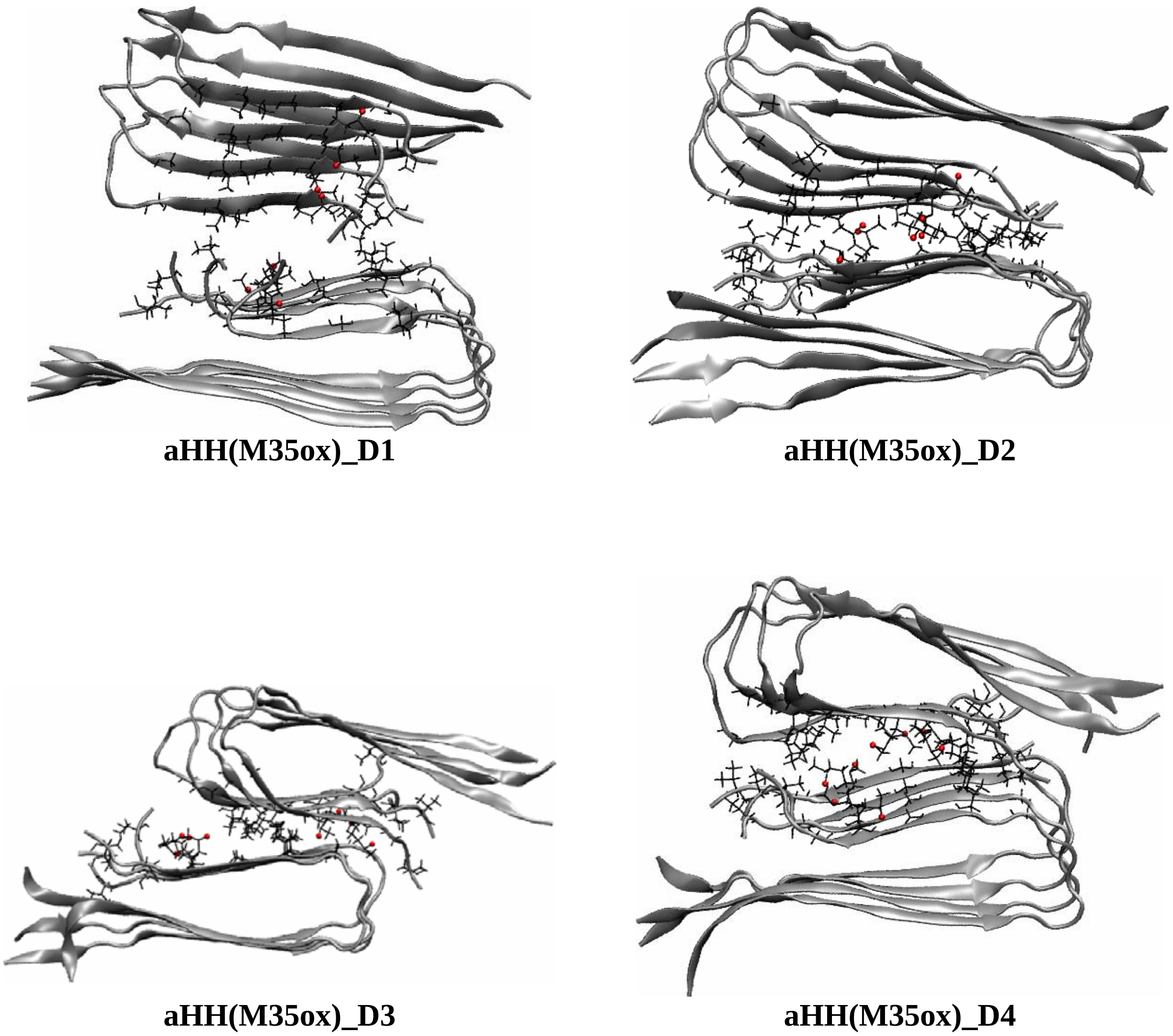

Figure S4 The last snapshots from 4 trajectories of construct aHH(M35ox) (see Table 1). Only inner 4 strands of a subunit are shown. Negatively charged, positively charged, polar and hydrophobic residues are colored red, blue, green and black, respectively. The oxygen of M35 is shown as a red VDW ball.

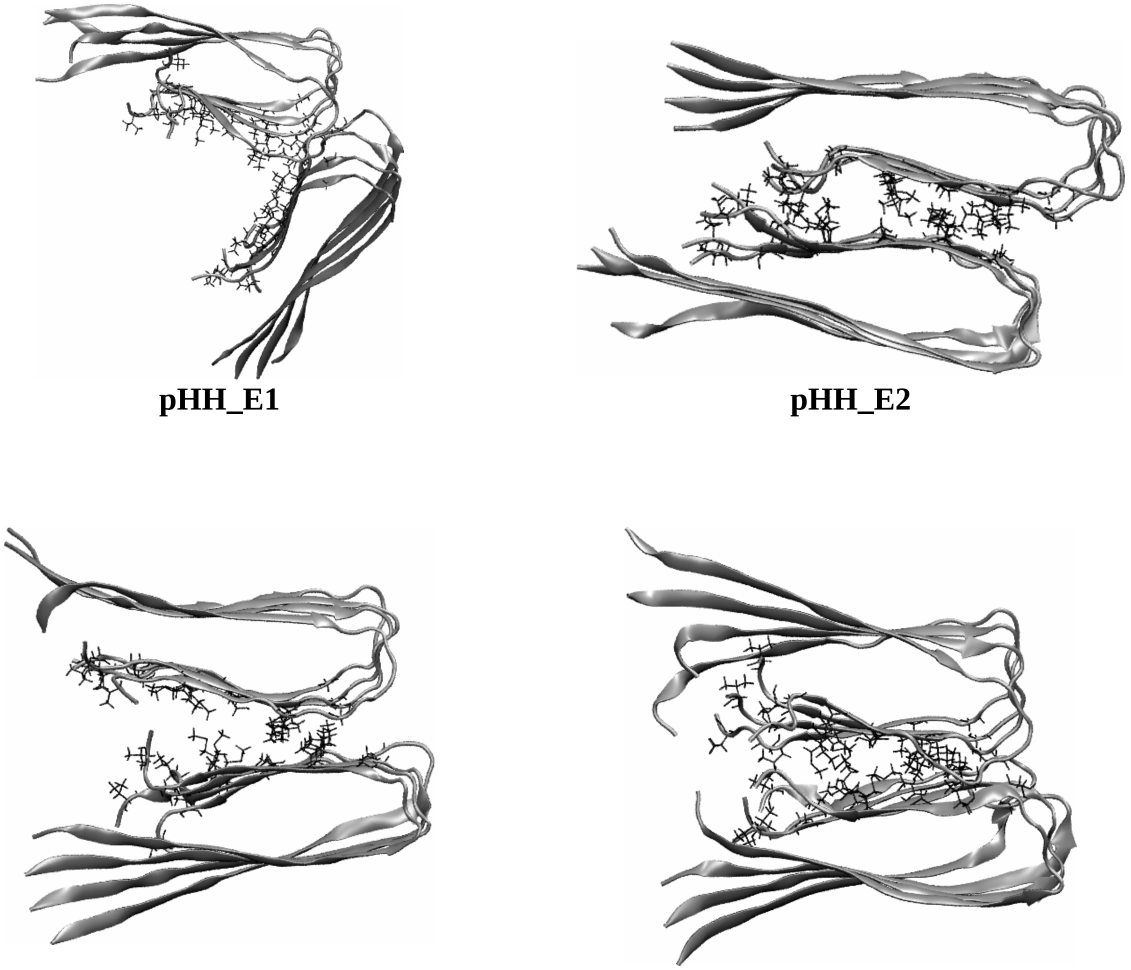

Figure S5 The last snapshots from 4 trajectories of construct pHH (see Table 1). Only inner 4 strands of a subunit are shown. Negatively charged, positively charged, polar and hydrophobic residues are colored red, blue, green and black, respectively.

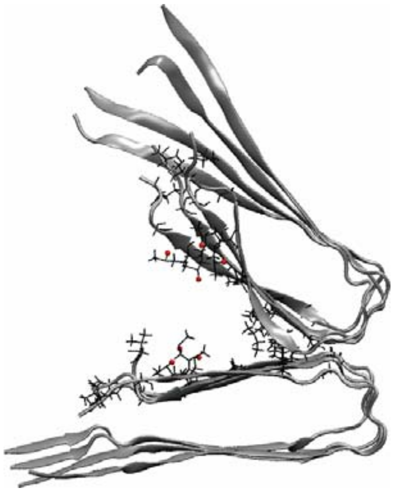

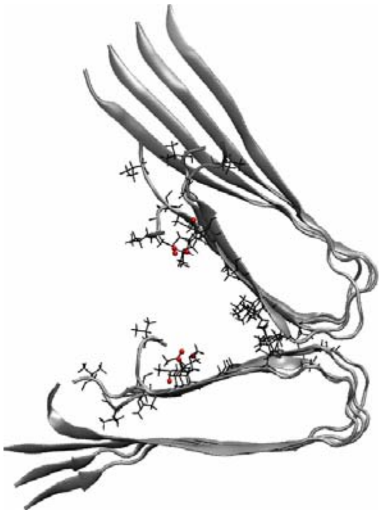

pHH(M35ox)_F1 **pHH(M35ox)_F2**

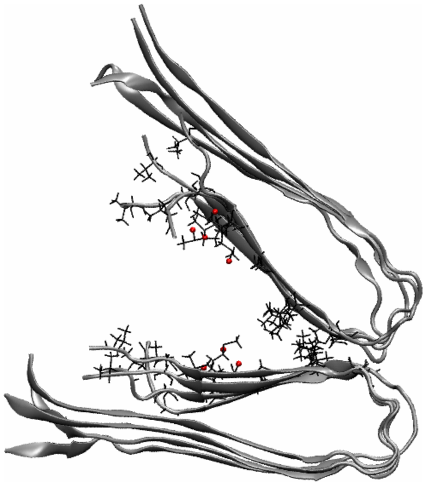

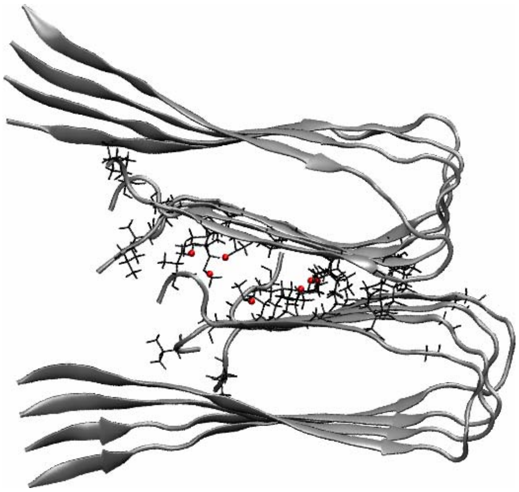

pHH(M35ox)_F3 **pHH(M35ox)_F4**

Figure S6 The last snapshots from 4 trajectories of construct pHH(M35ox) (see Table 1). Only inner 4 strands of a subunit are shown. Negatively charged, positively charged, polar and hydrophobic residues are colored red, blue, green and black, respectively. The oxygen of M35 is shown as a red VDW ball.

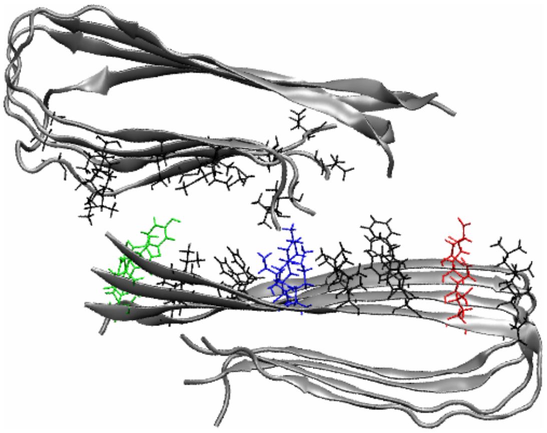

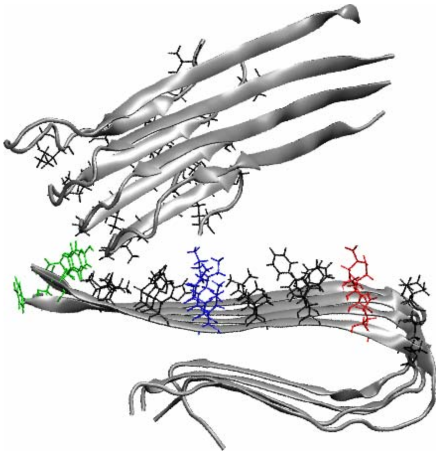

aHP_G1 **aHP_G2**

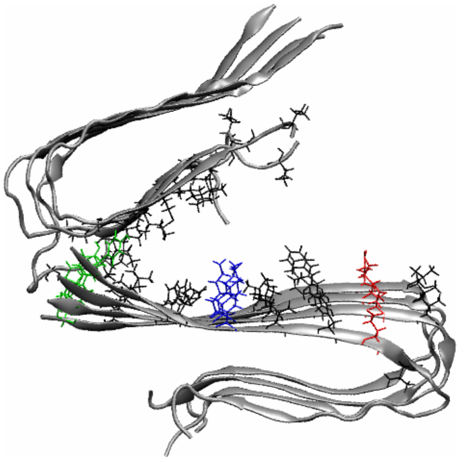

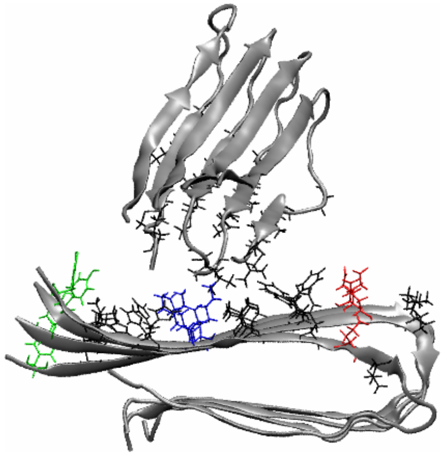

aHP_G3 **aHP_G4**

Figure S7 The last snapshots from 4 trajectories of construct aHP (see Table 1). Only inner 4 strands of a subunit are shown. Negatively charged, positively charged, polar and hydrophobic residues are colored red, blue, green and black, respectively.

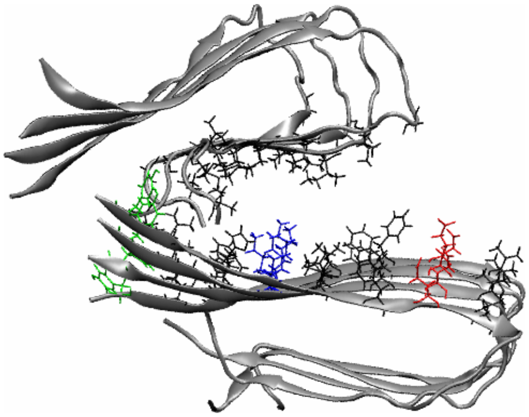

pHP_H1

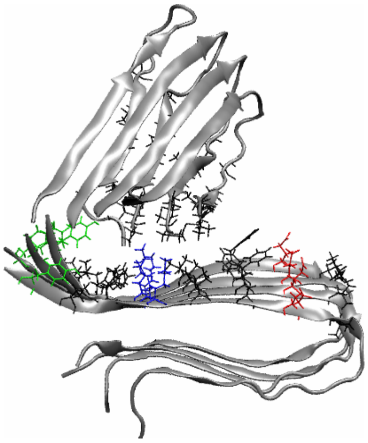

pHP_H2

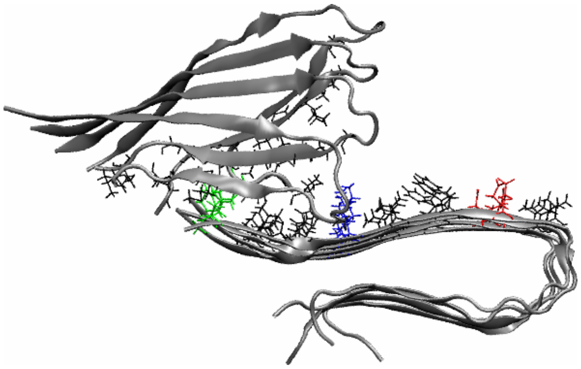

pHP_H3

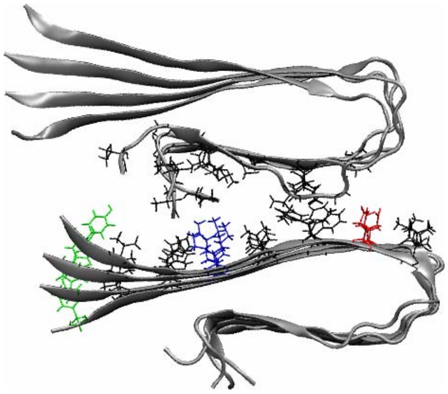

pHP_H4

Figure S8 The last snapshots from 4 trajectories of construct pHH (see Table 1). Only inner 4 strands of a subunit are shown. Negatively charged, positively charged, polar and hydrophobic residues are colored red, blue, green and black, respectively.

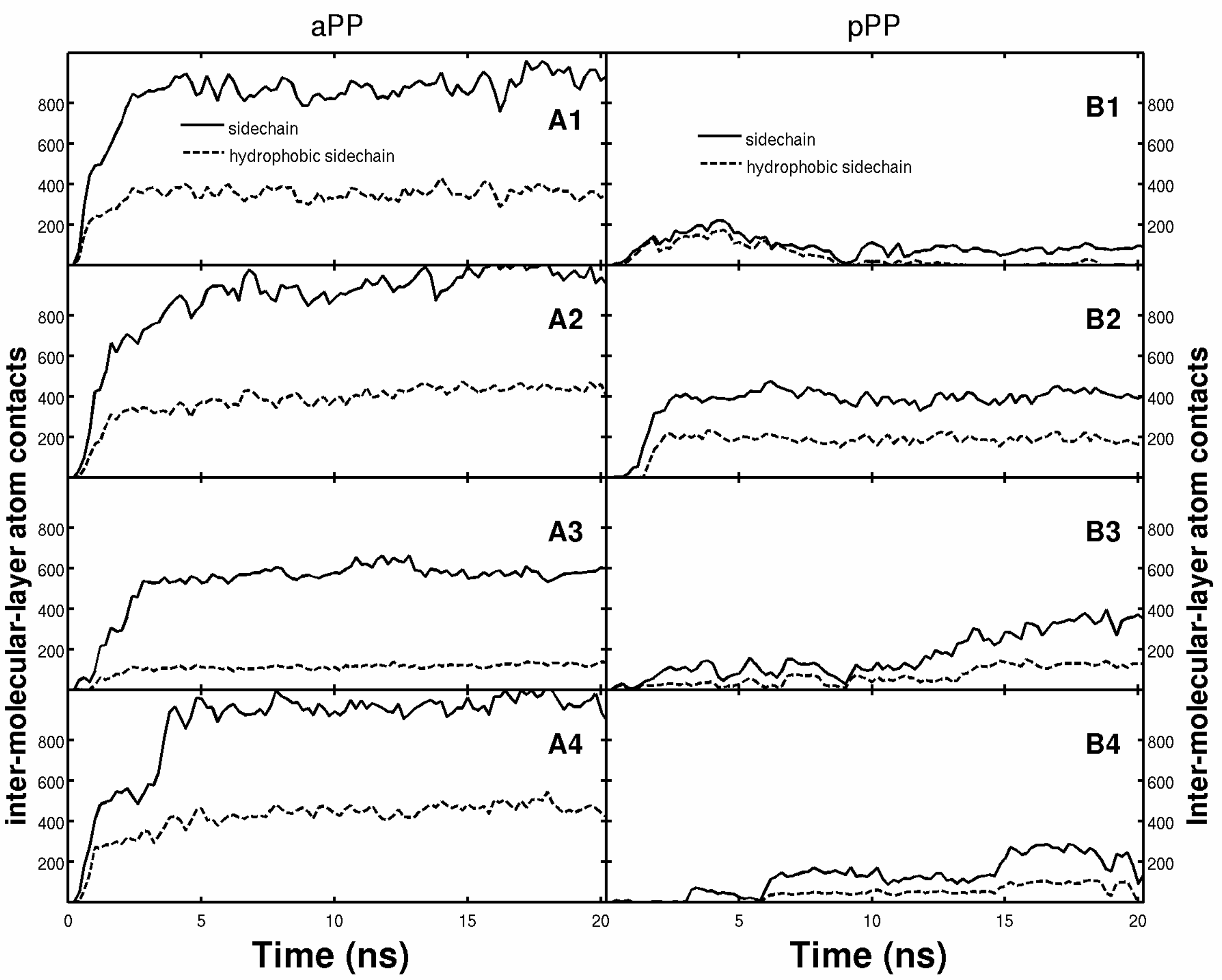

Figure S9 Development of side chain atom contacts (cutoff 4Å) between two cross-β subunits in constructs: aPP (trajectories A1-A4) and pPP (trajectories B1-B4).

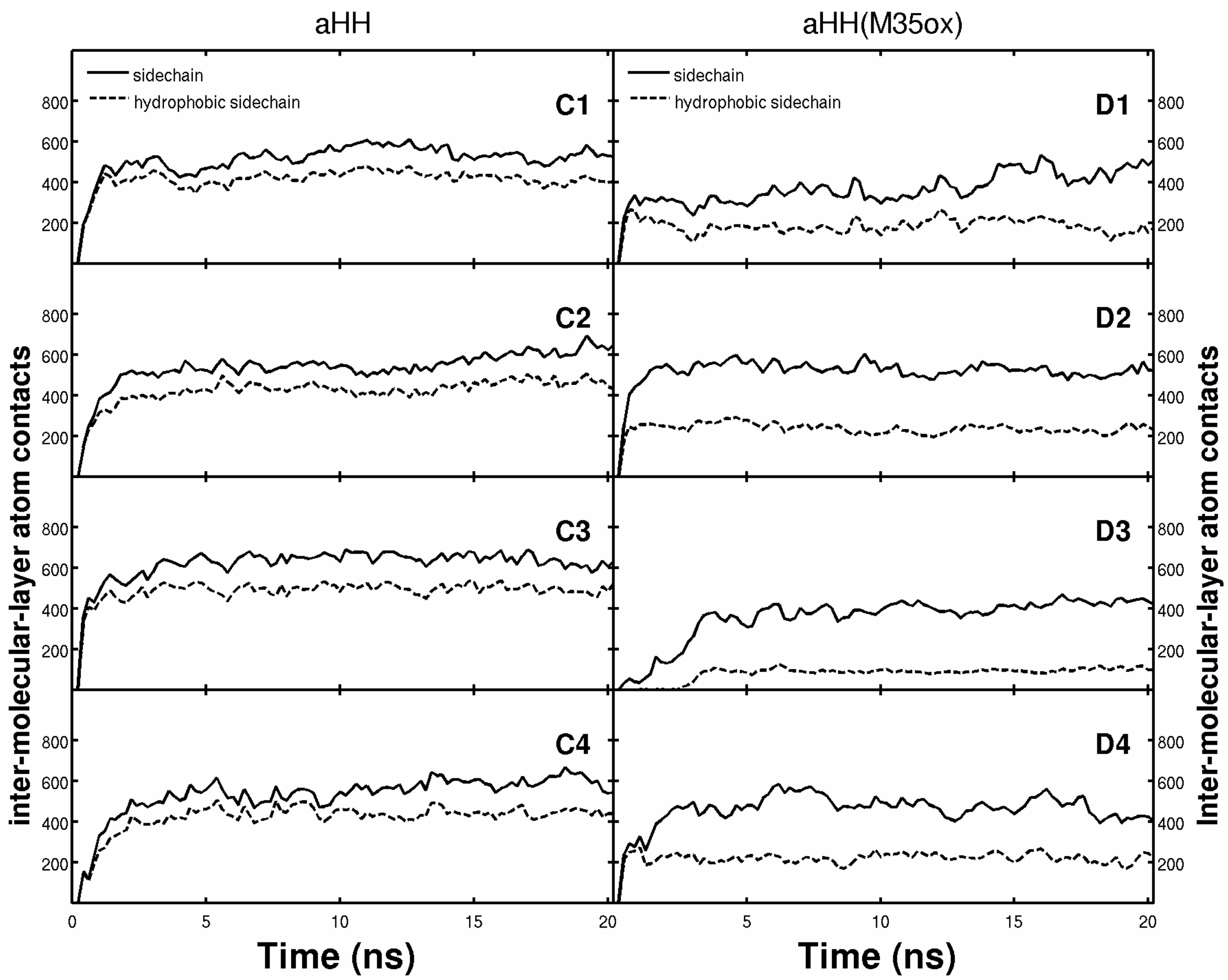

Figure S9 Development of side chain atom contacts (cutoff 4Å) between two cross-β subunits in constructs: aHH (trajectories C1-C4) and aHH(M35ox) (trajectories D1-D4).

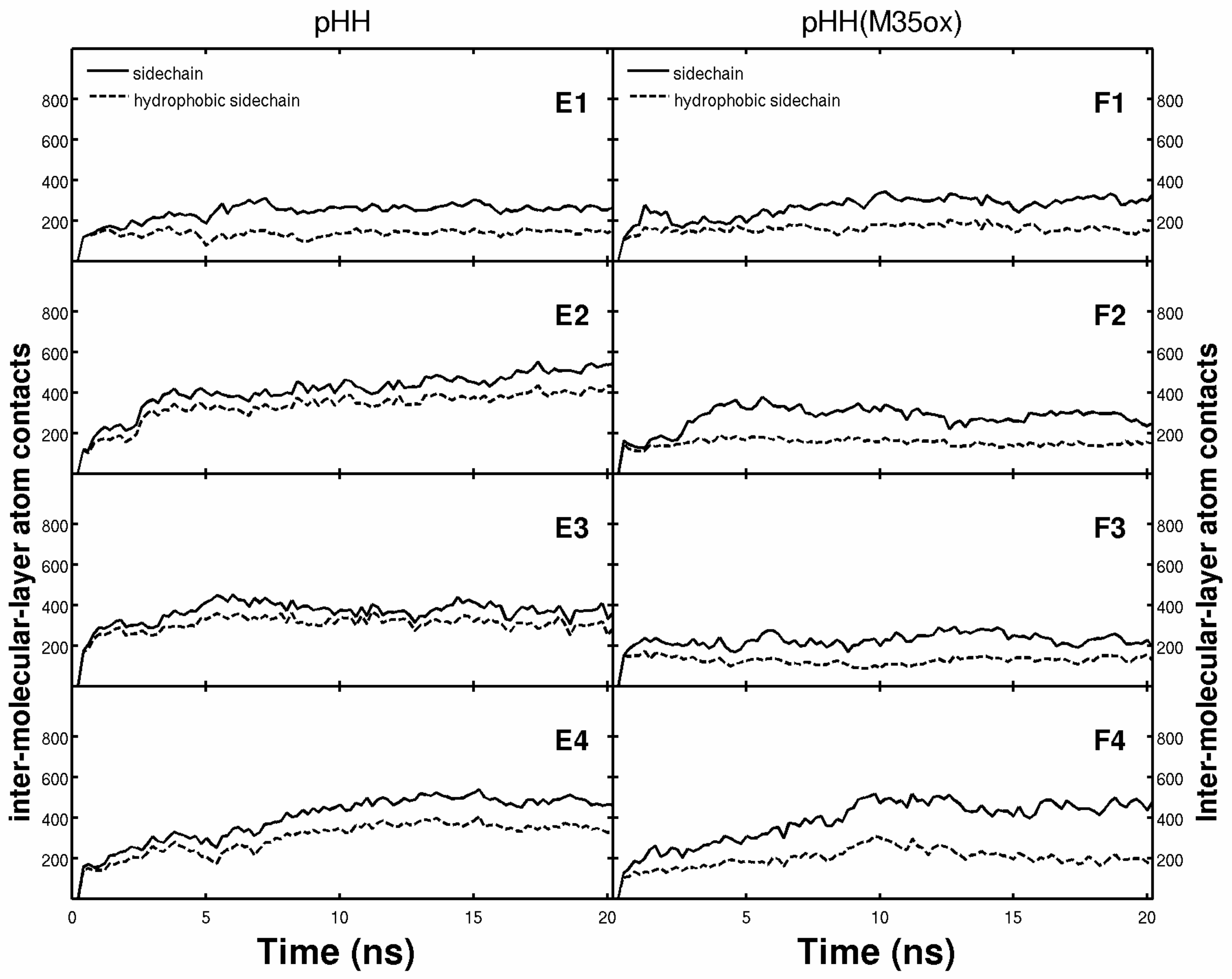

Figure S11 Development of side chain atom contacts (cutoff 4Å) between two cross-β subunits in constructs: pHH (trajectories E1-E4) and pHH(M35ox) (trajectories F1-F4).

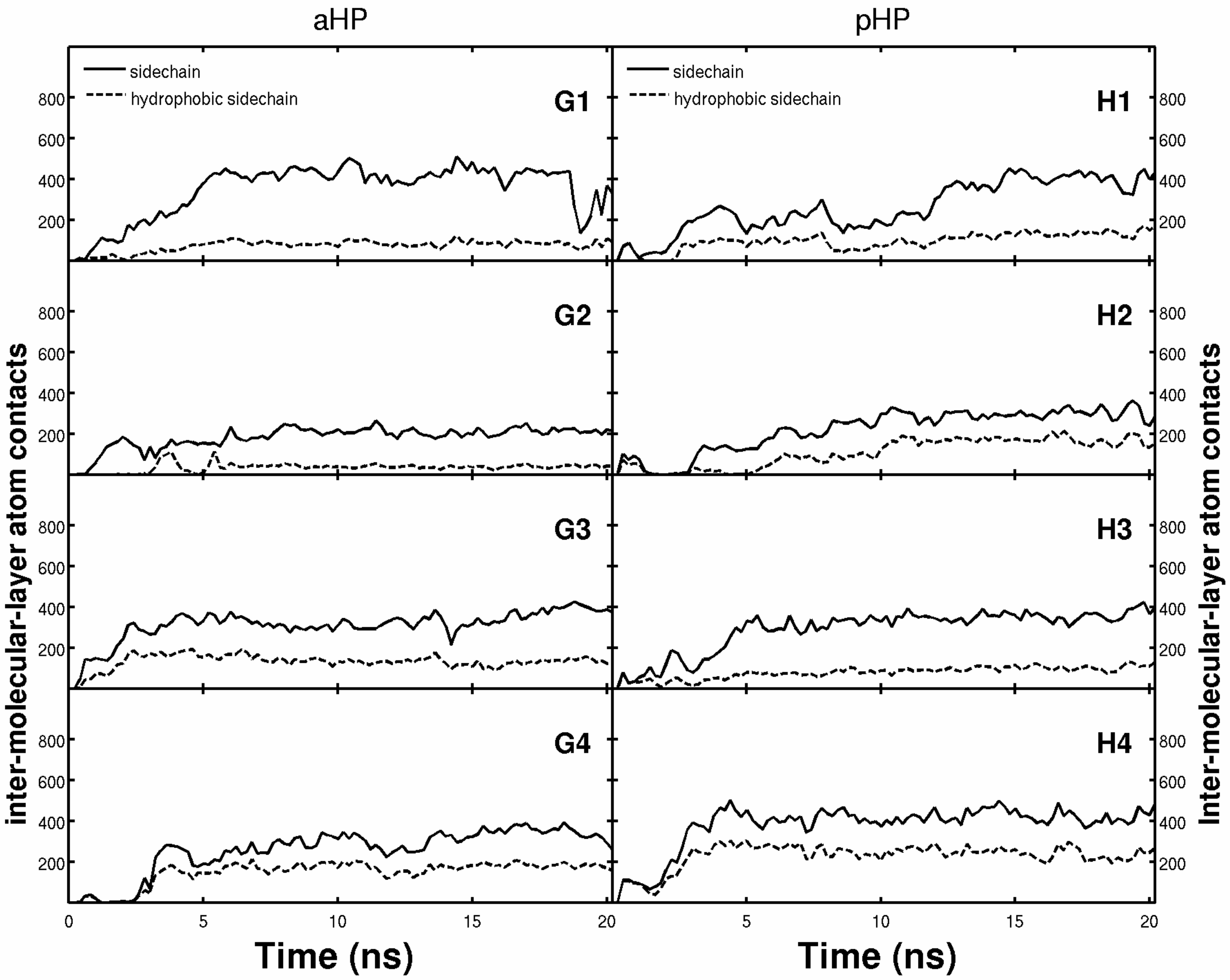

Figure S12 Development of side chain atom contacts (cutoff 4Å) between two cross-β subunits in constructs: aHP (trajectories G1-G4) and pHP (trajectories H1-H4).

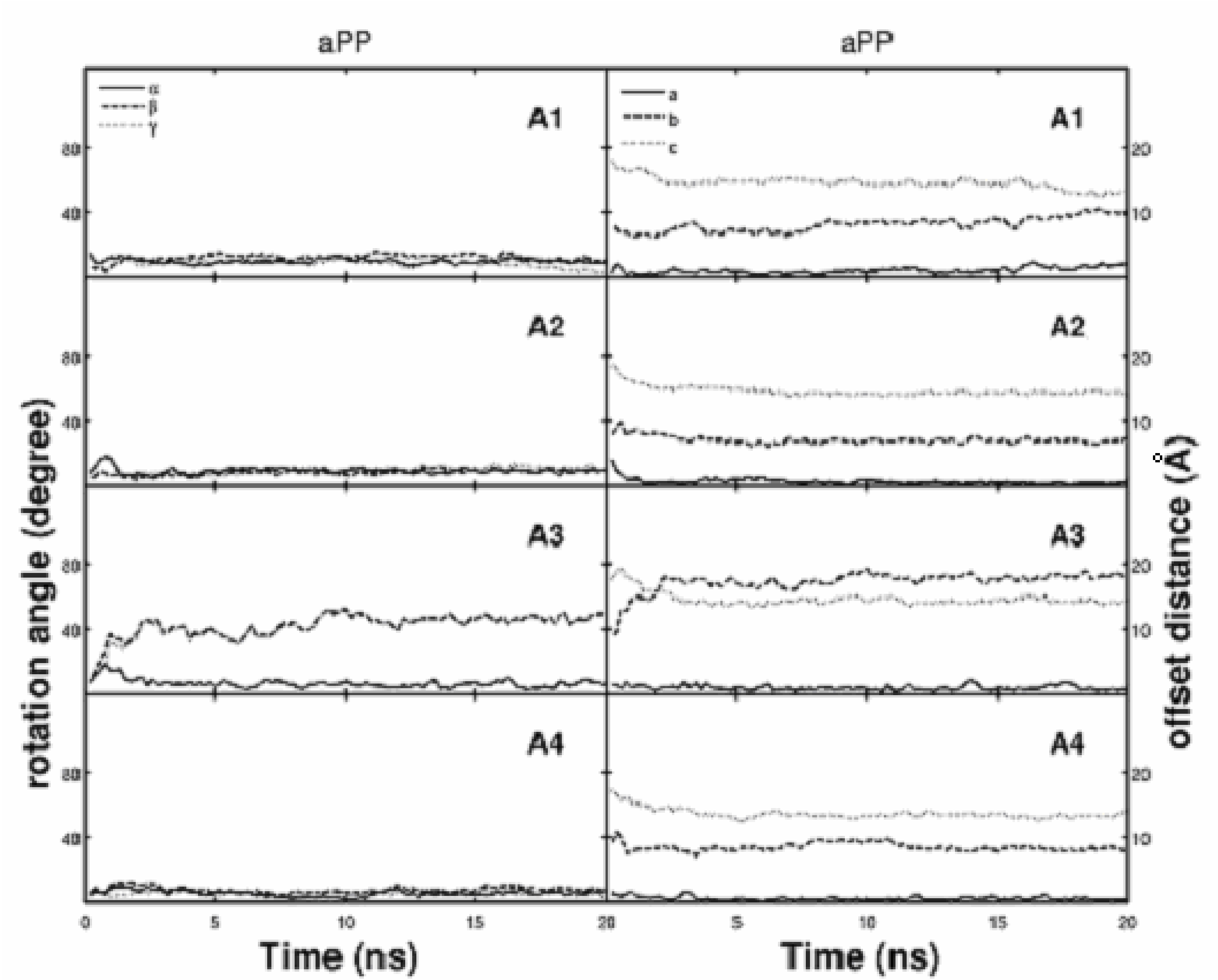

Figure S13 Development of 6 order parameters between two cross-β subunits of construct aPP (trajectories A1-A4). Six parameters (α, β, γ, a,b,c) are used to characterize the structural relationship (rotation and translation) between two interfacing β-sheets under a rigid body assumption. Left panel: (α, β, γ) are the rotation angles of the hydrogen bond direction (fibril axis), β-strand direction and β-sheet stacking direction, respectively. Right panel: (a, b, c) are translation distances along the hydrogen bond direction (fibril axis), β-strand direction and β-sheet stacking direction, respectively.

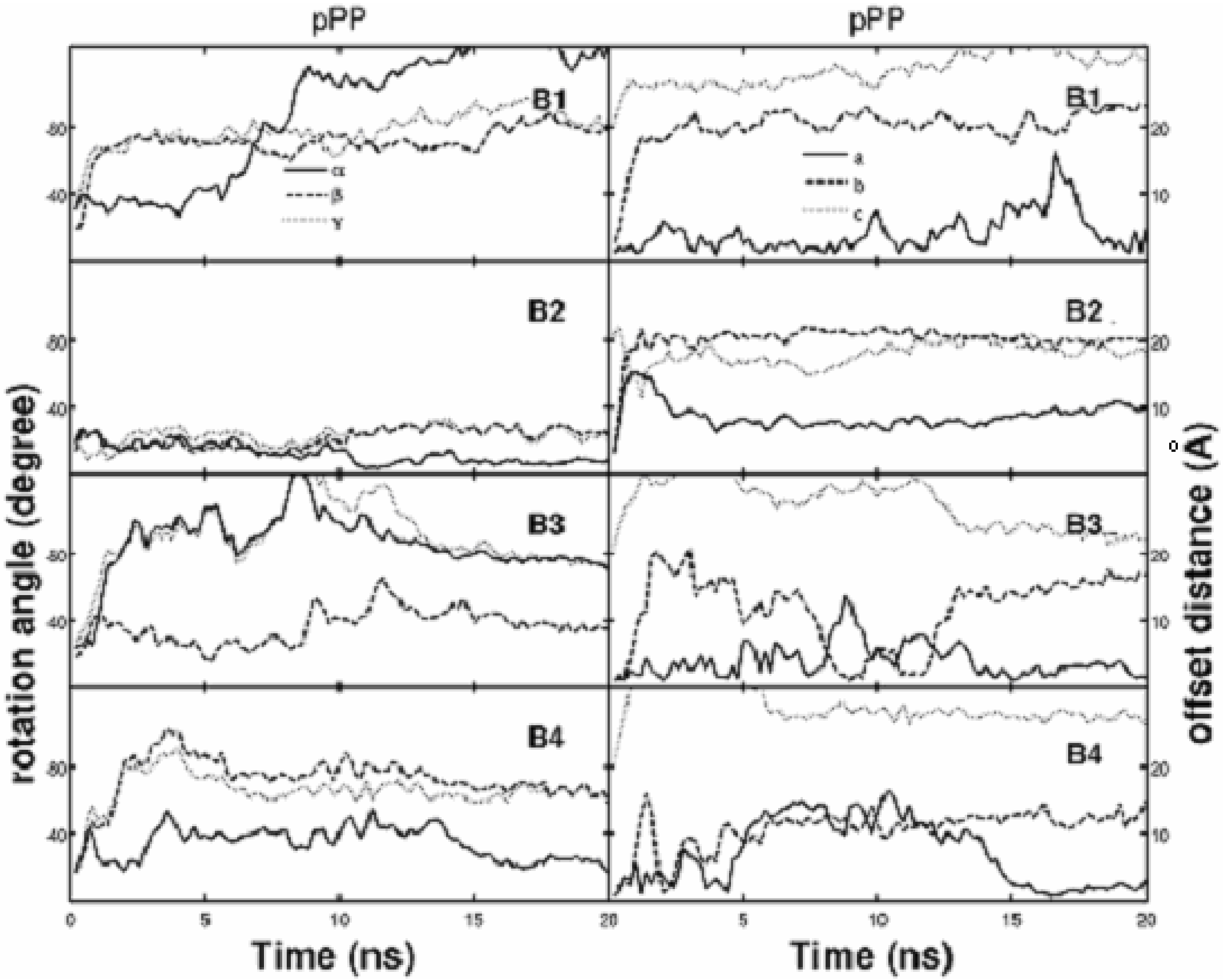

Figure S14 Development of 6 order parameters between two cross-β subunits in construct pPP(trajectories B1-B4). Left panel: (α, β, γ) are the rotation angles of the hydrogen bond direction (fibril axis), β-strand direction and β-sheet stacking direction, respectively. Right panel: (a, b, c) are translation distances along the hydrogen bond direction (fibril axis), β-strand direction and β-sheet stacking direction, respectively.

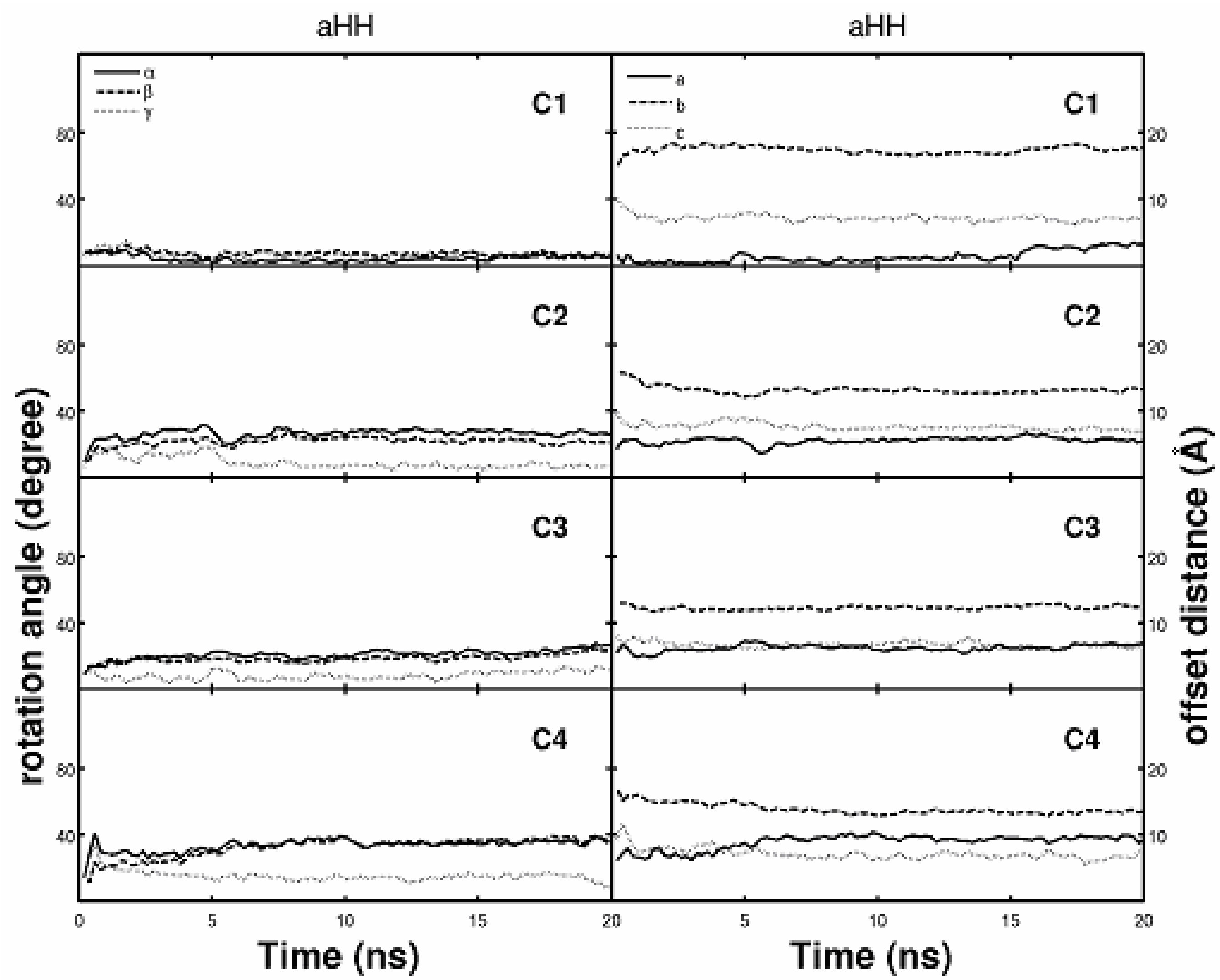

Figure S15 Development of 6 order parameters between two cross-β subunits in construct aHH(C1-C4). Left panel: (α, β, γ) are the rotation angles of the hydrogen bond direction (fibril axis), β-strand direction and β-sheet stacking direction, respectively. Right panel: (a, b, c) are translation distances along the hydrogen bond direction (fibril axis), β-strand direction and β-sheet stacking direction, respectively.

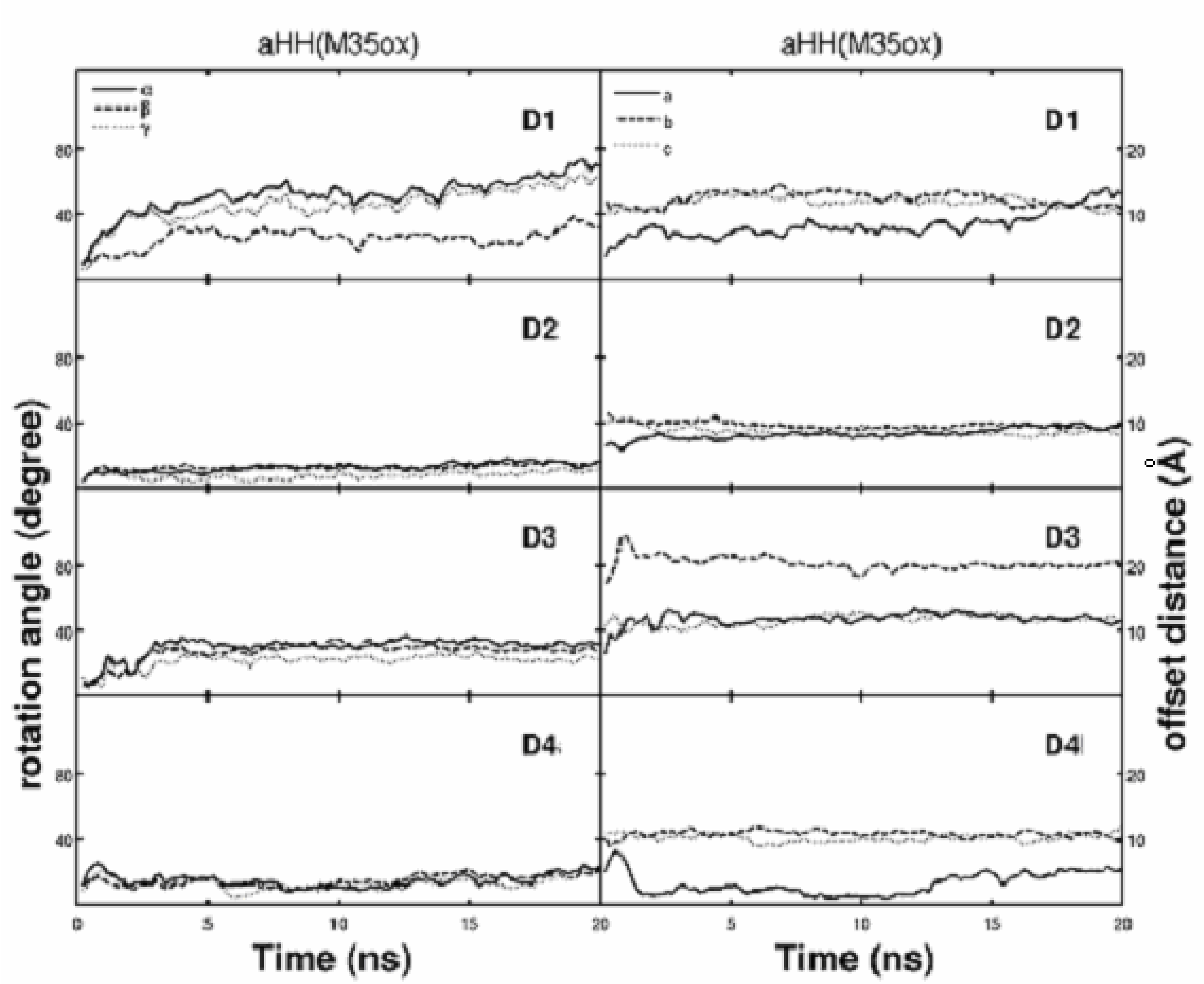

Figure S16 Development of 6 order parameters between two cross-β subunits in construct aHH(M35ox) (trajectories D1-D4). Left panel: (α, β, γ) are the rotation angles of the hydrogen bond direction (fibril axis), β-strand direction and β-sheet stacking direction, respectively. Right panel: (a, b, c) are translation distances along the hydrogen bond direction (fibril axis), β-strand direction and β-sheet stacking direction, respectively.

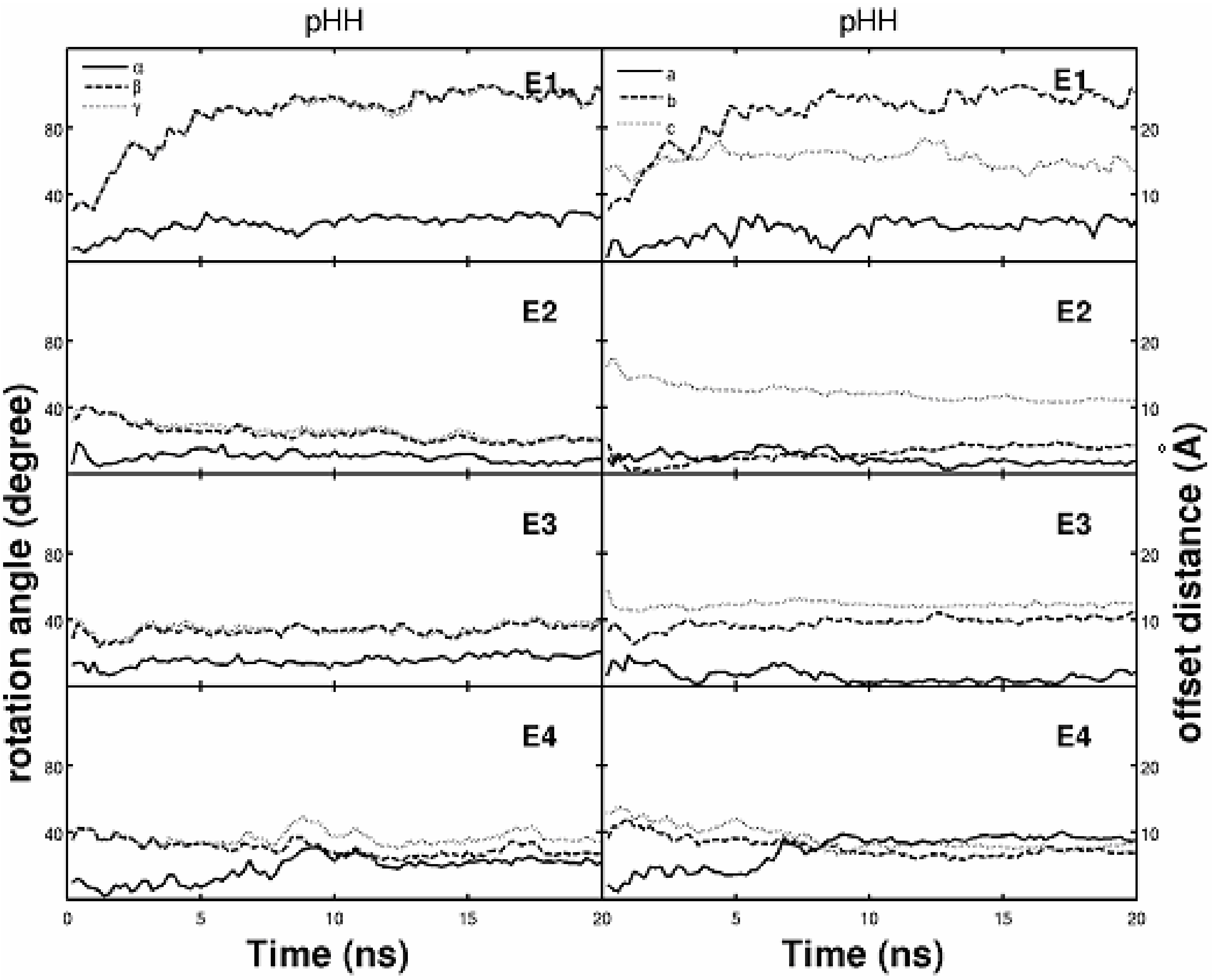

Figure S17 Development of 6 order parameters between two cross-β subunits of construct pHH (trajectories E1-E4). Left panel: (α, β, γ) are the rotation angles of the hydrogen bond direction (fibril axis), β-strand direction and β-sheet stacking direction, respectively. Right panel: (a, b, c) are translation distances along the hydrogen bond direction (fibril axis), β-strand direction and β-sheet stacking direction, respectively.

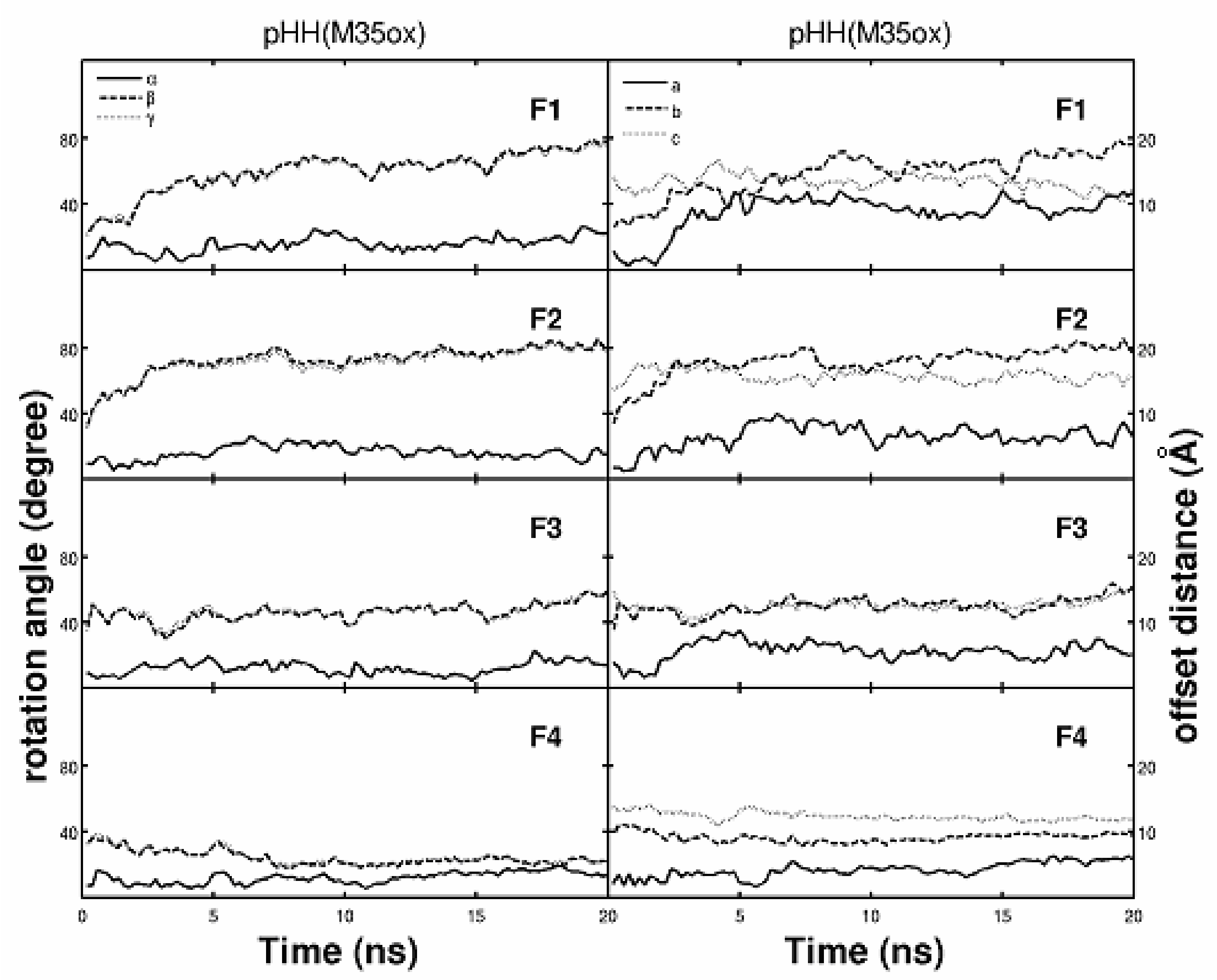

Figure S18 Development of 6 order parameters between two cross-β subunits in construct pHH(M35ox) (trajectories F1-F4). Left panel: (α, β, γ) are the rotation angles of the hydrogen bond direction (fibril axis), β-strand direction and β-sheet stacking direction, respectively. Right panel: (a, b, c) are translation distances along the hydrogen bond direction (fibril axis), β-strand direction and β-sheet stacking direction, respectively.

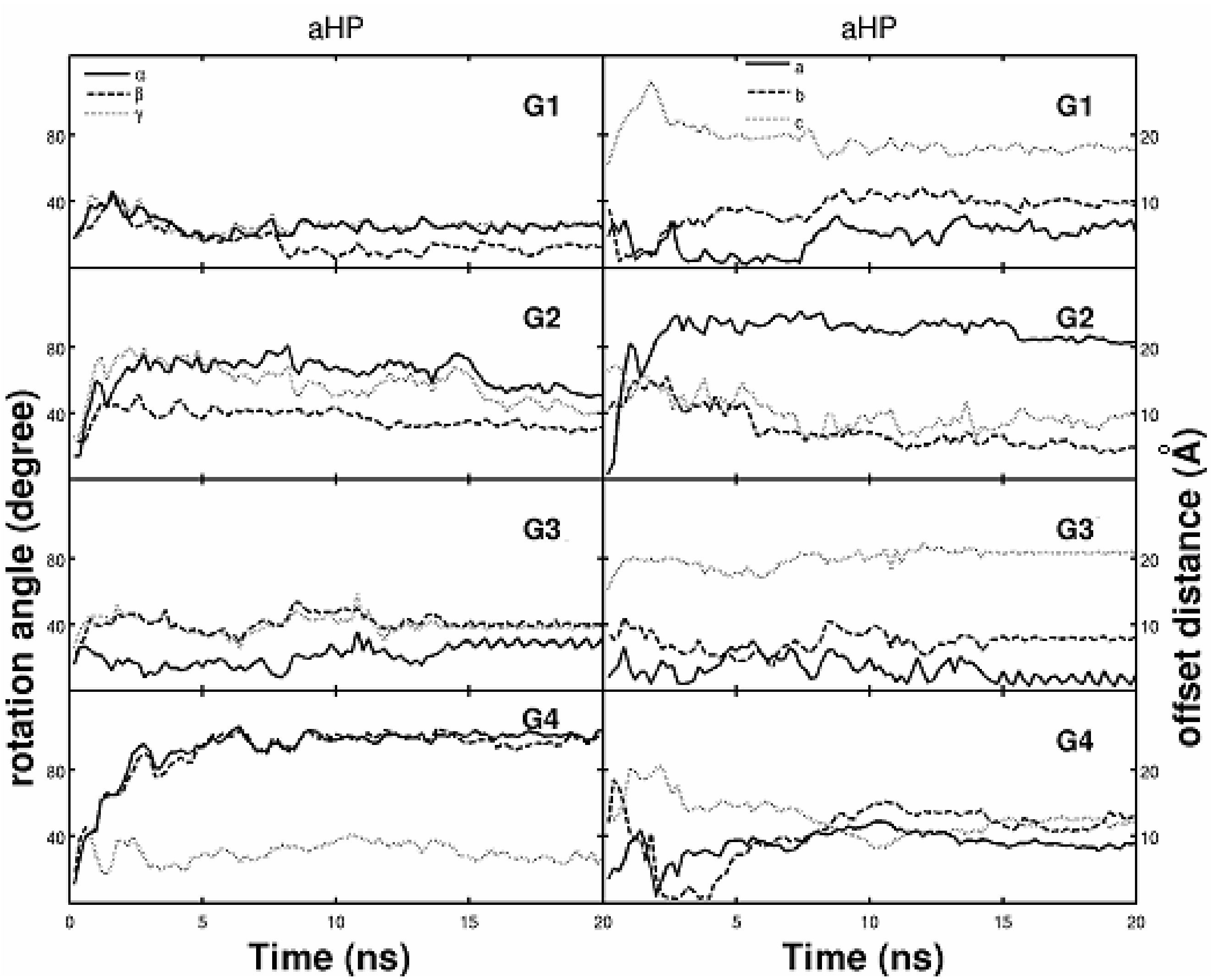

Figure S19 Development of 6 order parameters between two cross-β subunits in construct aHP(trajectories G1-G4). Left panel: (α, β, γ) are the rotation angles of the hydrogen bond direction (fibril axis), β-strand direction and β-sheet stacking direction, respectively. Right panel: (a, b, c) are translation distances along the hydrogen bond direction (fibril axis), β-strand direction and β-sheet stacking direction, respectively.

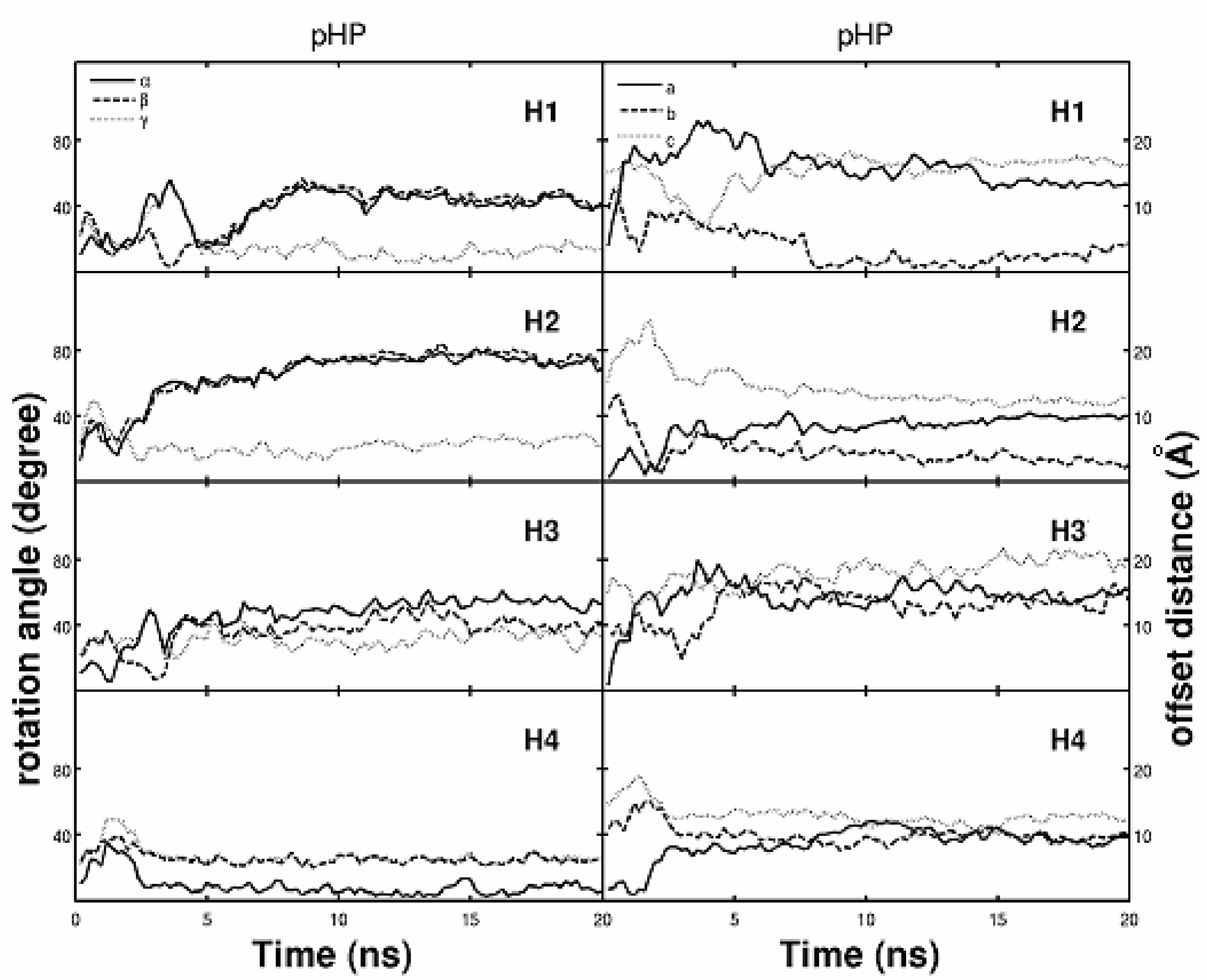

Figure S20 Development of 6 order parameters between two cross-β subunits in construct pHP(trajectories H1-H4). Left panel: (α, β, γ) are the rotation angles of the hydrogen bond direction (fibril axis), β-strand direction and β-sheet stacking direction, respectively. Right panel: (a, b, c) are translation distances along the hydrogen bond direction (fibril axis), β-strand direction and β-sheet stacking direction, respectively.

Figure S21 The binding energy over time.

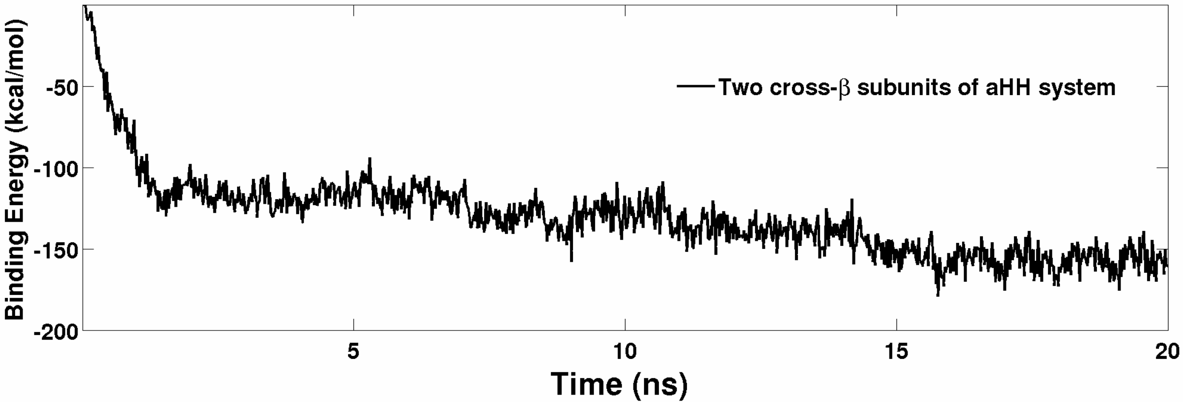

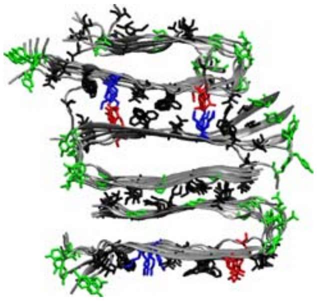

A1

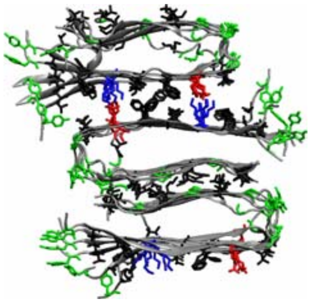

A2

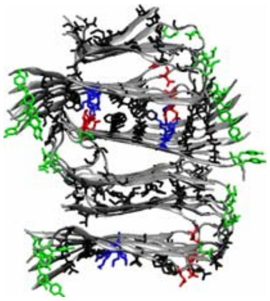

A2

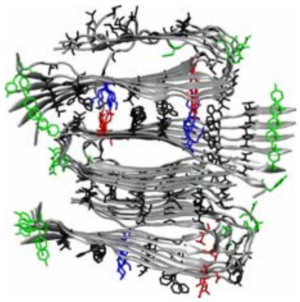

A3

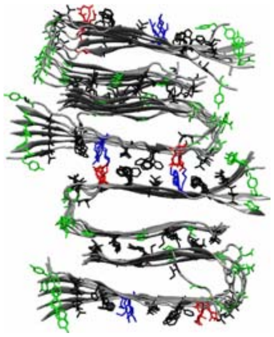

B1

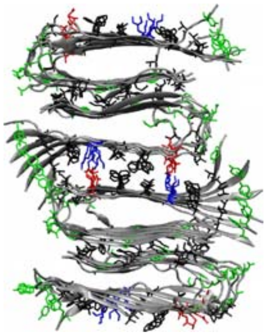

B2

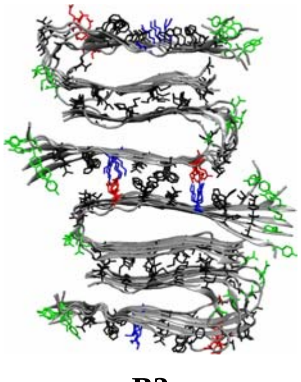

B3

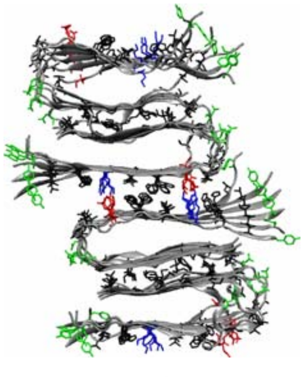

B4

Figure S22 Last snapshot of each stacking trajectory (4 trajectories for each construct) between two polar surfaces (aPP) at 310 K. A1-A4: “2+1” stacking (see Table 1). B1-B4: “2+2” stacking (see Table 1). Negatively charged, positively charged, polar and hydrophobic side chains are colored red, blue, green and black, respectively.

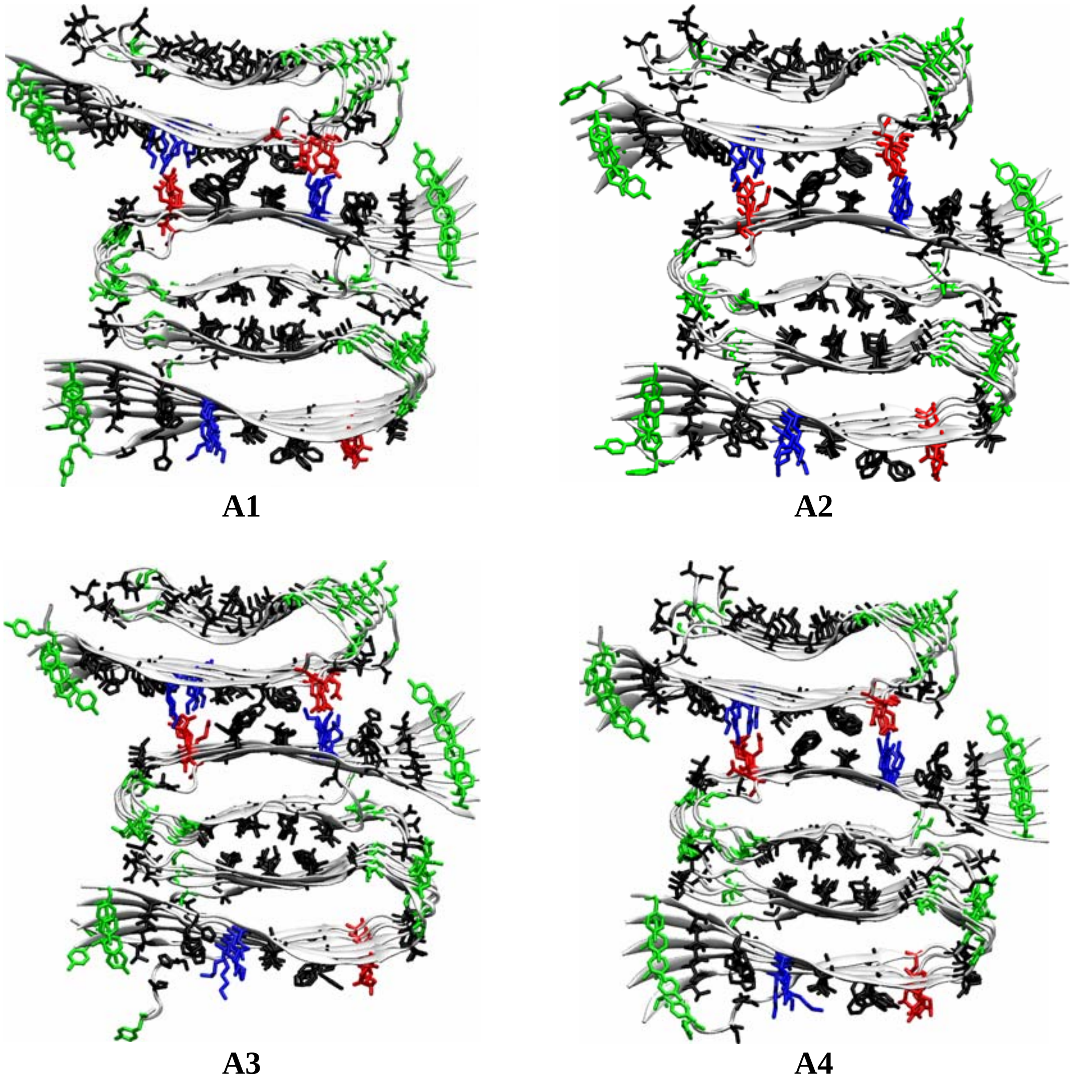

Figure S23 Last snapshot of 4 stability trajectories of brain-seed fibrils at 310 K. Negatively charged, positively charged, polar and hydrophobic side chains are colored red, blue, green and black, respectively.
